# Identifying Safeguards Disabled by Epstein-Barr Virus Infections in Genomes From Patients With Breast Cancer: Chromosomal Bioinformatics Analysis

**DOI:** 10.2196/50712

**Published:** 2025-01-29

**Authors:** Bernard Friedenson

**Affiliations:** 1 Department of Biochemistry and Medical Genetics, Cancer Center, University of Illinois Chicago, 900 s Ashland, Chicago, IL, 60617, United States, 1 8479124216

**Keywords:** breast cancer, cancer, oncology, ovarian, virus, viral, Epstein-Barr, herpes, bioinformatics, chromosome, gene, genetic, chromosomal, DNA, genomic, BRCA, metastasis, biology

## Abstract

**Background:**

The causes of breast cancer are poorly understood. A potential risk factor is Epstein-Barr virus (EBV), a lifelong infection nearly everyone acquires. EBV-transformed human mammary cells accelerate breast cancer when transplanted into immunosuppressed mice, but the virus can disappear as malignant cells reproduce. If this model applies to human breast cancers, then they should have genome damage characteristic of EBV infection.

**Objective:**

This study tests the hypothesis that EBV infection predisposes one to breast cancer by causing permanent genome damage that compromises cancer safeguards.

**Methods:**

Publicly available genome data from approximately 2100 breast cancers and 25 ovarian cancers were compared to cancers with proven associations to EBV, including 70 nasopharyngeal cancers, 90 Burkitt lymphomas, 88 diffuse large B-cell lymphomas, and 34 gastric cancers. Calculation algorithms to make these comparisons were developed.

**Results:**

Chromosome breakpoints in breast and ovarian cancer clustered around breakpoints in EBV-associated cancers. Breakpoint distributions in breast and EBV-associated cancers on some chromosomes were not confidently distinguished (*P*>.05), but differed from controls unrelated to EBV infection. Viral breakpoint clusters occurred in high-risk, sporadic, and other breast cancer subgroups. Breakpoint clusters disrupted gene functions essential for cancer protection, which remain compromised even if EBV infection disappears. As CRISPR (clustered regularly interspaced short palindromic repeats)–like reminders of past infection during evolution, EBV genome fragments were found regularly interspaced between Piwi-interacting RNA (piRNA) genes on chromosome 6. Both breast and EBV-associated cancers had inactivated genes that guard piRNA defenses and the major histocompatibility complex (MHC) locus. Breast and EBV-associated cancer breakpoints and other variations converged around the highly polymorphic MHC. Not everyone develops cancer because MHC differences produce differing responses to EBV infection. Chromosome shattering and mutation hot spots in breast cancers preferentially occurred at incorporated viral sequences. On chromosome 17, breast cancer breakpoints that clustered around those in EBV-mediated cancers were linked to estrogen effects. Other breast cancer breaks affected sites where EBV inhibits JAK-STAT and SWI-SNF signaling pathways. A characteristic EBV-cancer gene deletion that shifts metabolism to favor tumors was also found in breast cancers. These changes push breast cancer into metastasis and then favor survival of metastatic cells.

**Conclusions:**

EBV infection predisposes one to breast cancer and metastasis, even if the virus disappears. Identifying this pathogenic viral damage may improve screening, treatment, and prevention. Immunizing children against EBV may protect against breast, ovarian, other cancers, and potentially even chronic unexplained diseases.

## Introduction

In the United States, over 40,000 women die from breast cancer each year [1,2]. The causes of the disease are not well understood, making prevention and treatment empirical and hazardous. At the time of breast cancer diagnosis, its causes are difficult to isolate from multiple risk factors. A human cancer virus is one such risk factor. A tumor virus does not cause cancer by itself [3] but can make cancer more likely by inhibiting tumor suppressors [4] or activating oncogenes. Viral damage then increases cancer risks via mutations and chromosome breaks. Epstein-Barr virus (EBV), also called human herpesvirus 4, infects at least 90% of humans as a lifelong infection, often acquired at an early age [5], but the virus remains latent and asymptomatic in most people. EBV may be a risk factor for breast cancer. Active infection is significantly more prevalent in breast cancer tissues than in normal and benign controls [6], increasing risk by 4.75- to 6.29-fold [7]. EBV transformed human mammary epithelial cells in culture so that xenografts in immunosuppressed mice accelerated breast cancer. Once malignant transformation occurred, EBV was no longer required [8], but the cells remain malignant.

There has been no way to test the idea that EBV causes breast cancer and can then disappear. However, cancers in other tissues have proven relationships to EBV infection, so these known EBV-associated cancers can be compared to breast cancers at the genome level. Cancers with unambiguous EBV associations include nasopharyngeal cancer (NPC), EBV-positive diffuse large B-cell lymphoma (DLBCL), endemic Burkitt lymphoma (BL) [9], and gastric cancer (GC). Some genomic similarities between these EBV-associated cancers and breast cancer can be derived from the literature. In NPC, 100% of malignant cells are EBV positive [10]. Over 64% of NPCs are deficient in a pathway that depends on the breast cancer susceptibility genes *BRCA1* and *BRCA2* [11], which accurately repair DNA crosslinks and breaks via the homologous recombination pathway. This sprawling, interconnected pathway includes Fanconi anemia (FA) gene products and is often designated as the FA-BRCA pathway. In 126 patients with NPC, *BRCA1* and *BRCA2* were the most frequently mutated genes (55.5% and 33.3%, respectively) [12]. NPC mutations interfere with innate immunity and constitutively activate an inflammatory response. Overexpressed nuclear factor–κB (NF-κB) is a hallmark of NPC, occurring in 90% of NPCs [11]. Similarly, almost all stage-3 breast cancers overexpress NF-κB [13].

In NPC and the other known EBV-associated cancers, EBV inhibits the FA-BRCA pathway by various methods, including using viral microRNAs to downregulate *BRCA1* [14], hijacking other pathway components [15,16], and destabilizing SMC5/6-mediated chromatin interactions [17,18]. In GC, EBV infection and FA-BRCA pathway status are mutually exclusive [19], implying that EBV infection is approximately equivalent to disabling the FA-BRCA pathway. In DLBCL, the best prognostic marker is FA-BRCA pathway status [20]. In DLBCL and endemic BL, EBV variant infection accompanies *MYC* translocations. These translocations drive the disease and make a characteristic replacement of normal *MYC* control elements with highly active immunoglobulin regulatory sequences [21,22]. *MYC* amplification is frequent in breast cancers that have inactive *BRCA1* [23].

NPCs, DLBCLs, BLs, GCs, and breast cancers all have deficits in correctly repairing double-strand breaks and crosslinks. The compromised FA-BRCA pathway can produce chromosomes with too many centromeres. During cell division, mitotic spindles pull chromatids with multiple centromeres in too many directions, generating chromosome breaks to destabilize the human genome [24,25]. In breast cancer, these variations mark breakpoints at translocations and oncogene amplifications [26].

If EBV contributes to breast cancer, gene deficits in breast cancers and EBV-associated cancers should produce comparable changes in the human genome that do not depend on whether EBV infection persists. The aim of this study was to test for these virus-induced genome changes using bioinformatic calculations and analyses. The results could implicate EBV and its variants in disabling a variety of molecular and cellular safeguards that protect against breast cancer and its metastasis. Whether or not cancer develops in response to EBV infection depends on major histocompatibility complex (MHC) gene polymorphisms [27,28], so not everyone infected with EBV will develop cancer. In susceptible people, genome damage is permanent and does not require large numbers of viral particles, active infection, or continuing virus presence. Childhood immunization against selected EBV gene products may do much to prevent breast, ovarian, and other cancers.

## Methods

### Datasets Used in the Analysis

#### Overview

The initial data for analysis came from literature searches for studies on breast and EBV-associated cancers with large numbers of participants, unrestricted access to genome information, and complete whole-genome analysis. The first criterion for including breast cancer data was published intrachain or interchain chromosome breakpoints from high-quality, peer-reviewed publications produced by world-class laboratories. The second criterion was the availability of sufficient DNA sequence data to specify the location of these chromosome breakpoints. The third criterion was that genome sequencing had been done on samples taken before treatment began. These publicly available DNA sequence data were chosen to encompass diverse genetics, subtypes, stages, grades, morphologies, and outcomes. Initially, breast cancers were separated only broadly into those with a likely hereditary component versus those without this component. The cancers had to include typical morphologies such as ductal carcinomas, lobular carcinomas, medullary carcinomas, and invasive carcinomas (ie, “no special type”). The included breast cancers were all primary stage-2 or stage-3 cancers. Although surgery usually removes these primary tumors, cells with only a few additional late mutations are responsible for seeding local recurrences or metastases, so primary and metastatic tumors are not very different [29]. Although the selected cancers are not a random sample representing all breast cancers [30], they are likely to have chromosome instability originating from diverse typical causes.

Specifically, the breast cancer data used came from 560 breast cancer genome sequences, familial cancer data from 78 patients, methylation data from 1538 breast cancers versus 244 controls, 243 triple-negative breast cancers, and 2658 human cancers [31-35]. Data also included 74 breast cancers from high-risk women who were typed as having *BRCA1*- or *BRCA2*-associated mutations or cancers diagnosed before the age of 40 years [36,37]. Another study of familial breast cancers contributed 65 familial breast cancers [33]. Gene breakpoints for many interchromosomal and intrachromosomal translocations and breakpoints were obtained from the COSMIC (Catalog of Somatic Mutations in Cancer) website, as curated from original publications or original articles and their supplemental information [31-33]. Multimedia Appendix 1 provides a glossary of the terms used in this paper.

#### Breakpoints in Breast Cancers From High-Risk Women

Hereditary cancers were taken as breast cancers from women with a typed high-risk *BRCA1* or *BRCA2* mutation diagnosed before the age of 70 years. Cancers from patients with onset before the age of 50 years were also included to add more data, since these women are at high risk for an inherited, cancer-associated mutation. These patient samples were chosen based on descriptions in published data defining the breast cancer cohorts [31,33].

#### Sporadic Breast Cancers

Sporadic breast cancers were taken as breast cancers diagnosed after the age of 70 years that did not have a known inherited mutation [31].

#### Breast Cancer Subgroups

Human epidermal growth factor receptor 2 (HER2)–positive and triple-negative breast cancer data used for subgroup analysis were from original publications [33] and the COSMIC website.

#### Exclusions

Male breast cancers were excluded.

#### Data Source for Ovarian Cancers

Data for breakpoints in ovarian cancers were downloaded from the COSMIC website. The cancers corresponded to “mixed adenosquamous ovarian carcinomas” and were arbitrarily taken from those with the largest number of structural variants. These cancers all had the prefix “AOCS-” with further identification numbers and *BRCA* mutation status in parentheses as follows: 170-1-8 (negative), 120-3-6 (*BRCA2*), 142-3-5 (negative), 139-1-5 (negative), 086-3-2 (negative), 147-1-1 (*BRCA1* and *BRCA2*), 094‐6-X (*BRCA1*), 094-1-1 (*BRCA1*), 088-3-8 (negative), 139-6-3 (*BRCA2*), 150-3-1 (negative), 116-1-3 (negative), 155-3-5 (*BRCA2*), 093-3-6 (negative), 034-3-8 (*BRCA1*), 091-3-0 (*BRCA1*), 139-19-0 (*BRCA2*), 170-3-5 (negative), 114-1-8 (negative), 064-3-3 (negative), 064-1-6 (negative), 106-1-1 (*BRCA1*), 152‐1-X (*BRCA1*), and 134-1-5 (unknown).

### Original Data Sources for Cancers With Known EBV Associations: NPCs, Lymphomas, and GCs

#### Overview

NPC chromosome breakpoint positions were retrieved from Bruce et al [11] for 70 primary tumors of the nasopharynx at stages 1-4C. The data came from whole-genome sequencing of “63 micro-dissected tumors, 5-patient derived xenografts, and two cell lines.” DLBCL breakpoints were collected from 88 patients with DLBCL (aged >60 y) [22]. The *MYC* breakpoints included class I and II *MYC* translocation locus breakpoints defined in BL, encompassing areas far upstream of *c-myc* [38-40]. Downstream breakpoints included an enhancer region approximately 565 kilobases long on the nearest telomere side of the *MYC* coding sequence [22]. Older data provided fusion sequences as Gencode Accession numbers [21]. These fusion sequences were downloaded as FASTA files and copied to BLAST (Basic Local Alignment Search Tool) for placement on the human GRCh38/hg38 reference sequence. GCs with inferred EBV infection status came from 34 (20.2%) out of 168 samples subjected to whole-genome sequencing [41].

#### Selection Bias

As much as possible, selection bias was avoided by blindly selecting samples, replicating samples using cohorts from different publications, using the largest possible groups of samples, and avoiding convenience sampling. Some experiments used a newer dataset from 780 breast cancers [22] for comparisons to confirm that selection bias was unlikely.

#### Recruitment

Data from genome sequence studies did not include specific recruitment procedures for patients with cancer. However, patients are typically recruited through hospitals and clinics with referrals from medical professionals. Patients provide informed consent to have their genomes sequenced and used for research and to integrate cancer genome sequence data into treatment decisions [42].

### Methods Used to Determine That DNA Breakpoints From Breast and Ovarian Cancers Clustered Around Breakpoints in EBV-Associated Cancers

#### Calculation of Distances Between Breakpoints in Breast and Ovarian Cancers Versus EBV-Related Cancers

Before combining or comparing datasets, they were all converted to the same genome version, usually GRCh38. The break position in breast cancer nearest to a break in NPC was taken as the Microsoft Excel *XLOOKUP* value for the number of base pairs (bp) from the closest NPC breakpoint 5’ to the breast cancer break or the NPC breakpoint 3’ to the breast cancer break, whichever was closer (Multimedia Appendix 2). For comparing a given breast cancer breakpoint A2 to EBV-associated cancer breakpoints B2 to B72, the initial algorithm to find the nearest 5’ break position was written in cell C2 as follows:*=XLOOKUP($A2,$B$2:$B$72,$B$2:$B$72,0, −1,1)*. Changing −1 to +1 gave D2, the nearest 3’ position. Distance from the breast cancer breakpoint was then calculated as =*MIN(ABS(C2-A2), ABS(D2-A2))*. The same formulas were then continuously updated by Excel to calculate all other breast cancer comparisons in column A. Differences in the amount of data available for NPC versus breast cancer breakpoints complicated the calculations near chromosome telomeres. Several methods of handling these end regions made no discernible difference in the outcomes. For a 5000-bp window, an overflow window of 5,000,000 was used to limit the number of bins to a maximum of 1000. Another method of calculating distances between chromosome breakpoints in different cancers used the minimum of the absolute values of distances between breast cancers and the array of breakpoints in GCs, BLs, or NPCs. This method gave results identical to *XLOOKUP* values but was more convenient to compare clusters of breast cancer breakpoints to those in lymphoid and epithelial EBV-associated cancers. Hundreds of millions of calculations were repeated at least twice. Most of the calculations in this section are presented in Multimedia Appendix 2.

#### DNA Sequence Homology Analyses to Determine Breakpoints in Human Cancer Sequences That Resemble Viral Sequences

The NCBI BLASTn program (MegaBLAST) and database [43-45] were used to compare DNA sequence homologies around breakpoints in breast cancers to all available viral DNA sequences. *E* (“expect”) values are related to *P* values and represent the probability that a given homology bit score occurs by chance. *E* values <1×10^–10^ were considered significant homology. In many cases, *E* values were “0” (<1×10^–180^) and always far below 1×10^–10^. The virus DNA was retrieved from BLAST searches using “viruses (taxid:10239),” with human sequences, mouse sequences, and uncharacterized sample mixtures excluded. Different strains and isolates of the same virus were tested for human homology. Specifically, the HKHD40 and HKNPC60 variants were often considered together as “EBV.”

### Methods Used for Chromosome Comparisons of Breakpoints in Breast Cancers in High-Risk Women Versus Breakpoints in Sporadic Breast Cancer

The NCBI Genome Decoration page provided chromosome annotation software [46].

#### Identifying Genes Around the Most Frequent EBV-Binding Site Locations and Tethering Sites

EBV nuclear antigen 1 (EBNA1)–binding location genome coordinates [47,48] were used to tabulate genes within or near anchoring sites where EBV docks on human DNA. Breaks in breast cancers were compared to the gene positions around their EBNA1-binding sites. The Palindrome Site Finder from NovoPro and the EMBOSS palindrome program were used to identify palindromic DNA sequences.

#### Comparisons for Similarities Among Human Herpesviruses

EBV variants HKHD40 and HKNPC60 were compared to human herpesviruses in BLASTn by entering the terms “human gamma herpesvirus 4,” “herpesviridae,” and “herpesvirales.” Values with ≥2000 bp in common were selected. The EBV reference sequence was also tested against the following proven cancer viruses: human herpesvirus 8 (also called Kaposi sarcoma virus), herpes simplex virus 1, and human cytomegalovirus.

#### Locating Piwi-Interacting RNA Sequences as Evidence of Past EBV Infection

Piwi-interacting RNA (piRNA) locations were retrieved from the piRNA bank [49,50]. To compare the positions of piRNAs in virus homology versus genome position graphs, the midpoints of piRNA sequences were assigned arbitrary homology values. Positions of differentially methylated regions near breast cancer breakpoints on chromosome 6 [51] were compared to breakpoint positions for 70 NPCs based on published data analyses [11].

#### Viral Sequences in Human Genomes as Hypermutation and Rearrangement Sites in Breast Cancers

A graph of viral sequences in humans against chromothripsis breaks in breast cancers was so complex that it resisted interpretation, so only the 5 viral sequences nearest the chromothripsis breaks were used. The viral sequences nearest high-confidence chromothripsis breaks were determined in 5 iterations as genome coordinates where *XLOOKUP* values gave the minimum distances. Distances between all virus homology start points were then compared to all chromothripsis breakpoints.

### Methods of Data Analyses and Statistical Software

DNA flanking sequences at breakpoints were downloaded primarily from the GRCh38/hg38 version of the University of California, Santa Cruz Genome Browser as FASTA files and copied directly into BLAST. Results were checked against breakpoints in 101 triple-negative breast cancers from a population-based study [32]. The University of California, Santa Cruz Genome Browser’s *Liftover* function interconverted different versions of genome coordinates into GRCh38/hg38 coordinates.

#### Statistics

Excel, SPSS (IBM Corp), StatsDirect, Visual Basic (Microsoft), and Python (Python Software Foundation) scripts were used for data analysis. Mann-Whitney *U* tests compared overall breakpoint distributions [52] and tested the hypothesis that breakpoint distributions were identical or at least roughly the same. The Mann-Whitney *U* test was chosen because the comparisons involved unequal numbers of breakpoints, and each observation was likely independent. *P* values >.05 were taken to indicate that identical distributions could not be excluded. Tests for normality included kurtosis and skewness values and evaluation by Shapiro-Francia and Shapiro-Wilk methods [53] (Multimedia Appendix 2). The Fisher exact test compared breakpoints in breast cancers to those in known viral cancers. The unpaired 2-tailed Student *t* test was used to compare the means of numbers of breast cancers with severe versus nil lymphocyte infiltrates, assuming the data approximated normality and that there were no extreme outliers. Both of these tests require independence and random sampling. All these test results are only approximate because they depend on underlying assumptions.

#### Fragile Site Sequence Data

Positions of common fragile sites were retrieved from a database [54] and original publications [55].

### Ethical Considerations

This study presents analyses of publicly available data without recruiting additional human or animal subjects. Because this study is a secondary analysis, it is exempt from institutional review board and ethics approval. The data are in the public domain and are available for independent research and analysis [56]. It is not necessary to obtain permission to reuse public data. The original informed consent allows secondary analysis without additional permission.

## Results

### Breakpoints in Breast Cancers From High-Risk Backgrounds Clustered Around Breakpoints in NPC, an EBV-Mediated Cancer

EBV-mediated cancers such as NPC have defects in DNA repair and inflammatory pathways, resembling hereditary breast and ovarian cancers. To further characterize this resemblance, breakpoints in 70 NPC genomes were compared to breakpoints in 139 breast cancer genomes from high-risk women (*BRCA1*/*BRCA2* mutation, familial concentration, or young age).

**Figure 1.**
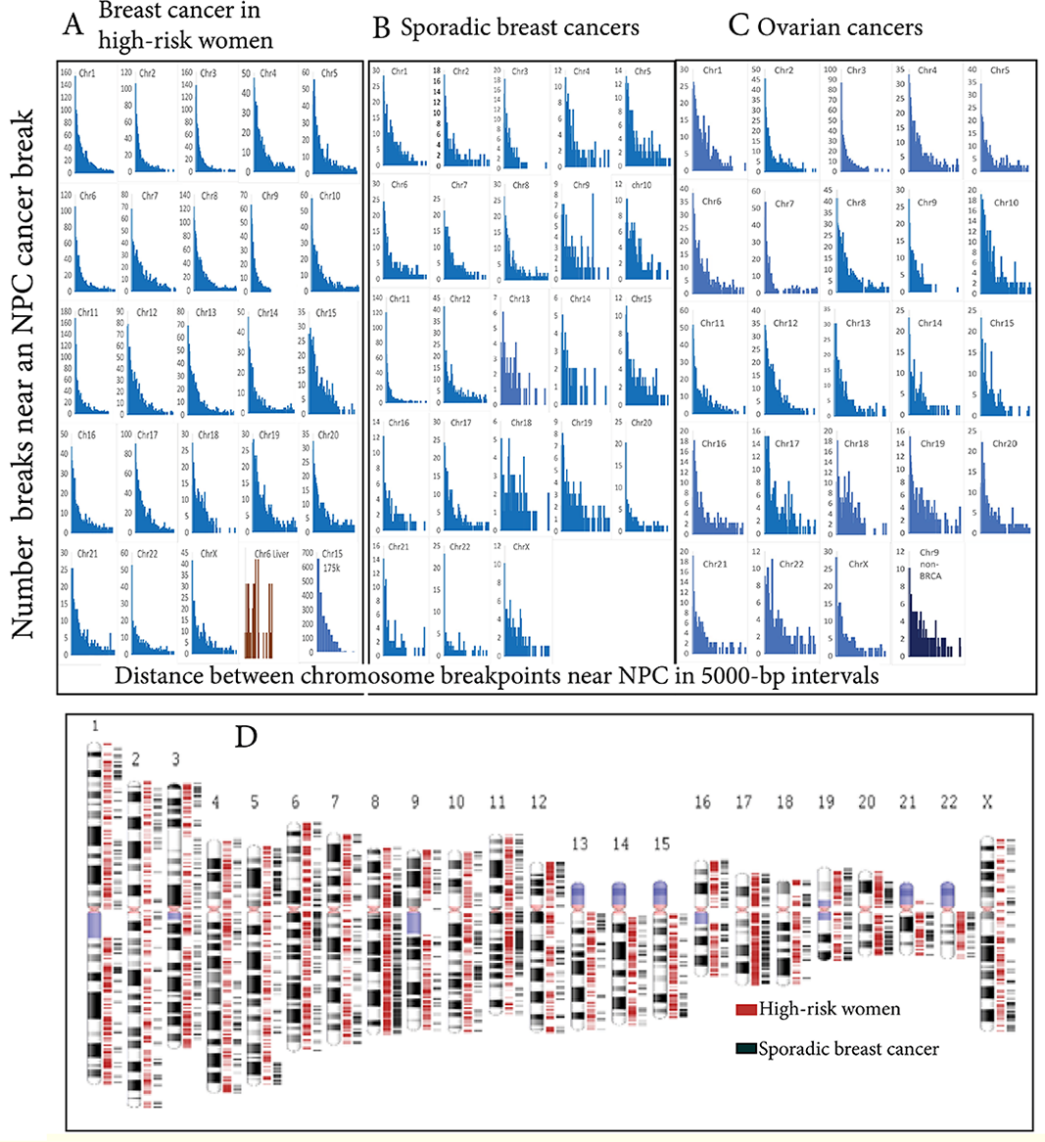
(A) Breakpoints in 139 breast cancers from high-risk women (*BRCA* mutation, familial concentration, or early onset) clustered around breakpoints in 70 NPCs. The data were grouped in 5000-bp increments to allow for methodological and laboratory differences. An unrelated set of hepatocellular data associated with hepatitis B insertions did not show a similar relationship to NPC. Breast cancer and NPC breakpoint distributions could not be confidently distinguished (*P*>.05) for chromosomes 6, 7, 10, 13, 14, 15, 22, and X (Multimedia Appendix 2). Many breakpoints were virtually the same on some chromosomes. The panel at the lower right shows how the selection of a larger bin size of 175,000 bp (the approximate length of EBV) affects the distributions of breakpoints. (B) Like the breast cancers from high-risk women, breakpoints in 74 sporadic breast cancers clustered around the breakpoints found in 70 NPCs. Breast cancer breakpoints within 5000 bp of an NPC breakpoint were the largest single category on most chromosomes. (C) Breakpoints in 25 mixed adenosquamous ovarian cancers also clustered around breakpoints in the 70 NPCs. The data show both *BRCA*-associated and nonassociated ovarian cancers. The panel in the lower right corner represents chromosome-9 data after removing all *BRCA*-associated ovarian cancers. The sporadic cancers show the same results as the complete set but with less data. (D) Many breakpoints in sporadic breast cancers clustered at chromosomal locations similar to those from high-risk women. Interchromosome translocation break positions in 74 mutation-associated, familial, or early-onset female breast cancers (red) versus 74 likely sporadic female breast cancers (black) are shown. bp: base pairs; Chr: chromosome; EBV: Epstein-Barr virus; NPC: nasopharyngeal cancer.

The distances from all breast cancer breakpoints to the nearest NPC breakpoints across the entire length of chromosome 1 produced results with so many points that they were difficult to interpret (Multimedia Appendix 3). Different laboratories collected these breakpoint data over many years. To allow for some variations, the data were grouped into 5000-bp increments (2×10^–5^ relative error). As shown in Figure 1 and Multimedia Appendix 2, breast cancer breakpoints were most often clustered within 5000 bp of NPC breakpoints, but many breakpoints agreed much more closely. A total of 20 breast cancer breakpoints on chromosome 1 were within 500 bp of an NPC breakpoint, and several chromosomes had breast cancer and NPC breakpoints in essentially the same positions. As represented by Mann-Whitney *U* test results (Multimedia Appendix 2), breast cancer and NPC breakpoint distributions were statistically the same (*P*>.05) for chromosomes 6, 7, 10, 13, 14, 15, 22, and X, but different on chromosome 1 and other chromosomes (*P*<.05).

In contrast, liver cancer breakpoints at hepatitis B virus integration sites [57] differed from those in breast cancer or NPC (Figure 1). No breaks in 114 liver cancers on chromosome 1 were within 5000 bp of breaks in any NPC; only one break on chromosome 6 in 61 liver cancers fit this window. According to a meta-analysis, the chance that breakpoints on chromosomes 1, 2, 6, and 8 were not within 5000 bp in liver cancer versus NPC was 4.4 (95% CI 1.9‐10). NPC and liver cancer did not have the same breakpoint distributions (*P*<.001).

The above results revealed that breast cancer breakpoints in high-risk women were clustered near those in the EBV-associated cancer, NPC, on every chromosome. The next step was to decide whether these similarities depended on mutations in the breast cancer susceptibility genes, *BRCA1* or *BRCA2*, by comparisons to sporadic breast cancers. The sporadic breast cancer group comprised 74 women, aged ≥70 years, with normal *BRCA* genes and no other known inherited, cancer-associated mutations [31]. Like breakpoints from high-risk women, many sporadic breast cancer breakpoints clustered around those in NPC (Figure 1). Breakpoints in these sporadic breast cancers clustered at chromosomal locations similar to breast cancers from high-risk women, although the frequencies and distributions sometimes differed significantly. The patients with sporadic breast cancer were older than the high-risk women, arguing against age as responsible for similarity to NPC breakpoints.

### Viral Homologies Around Breakpoints in Mixed Adenosquamous Ovarian Carcinoma Also Clustered Around Breakpoints in EBV-Mediated Cancer

Ovarian cancer data enabled an additional test for EBV involvement in breast cancer because, like breast cancer, *BRCA1* or *BRCA2* mutations can also predispose patients to ovarian cancer [58]. Chromosome breakpoints in 25 mixed adenosquamous ovarian cancers were compared to breakpoints in NPCs. The results depicted in Figure 1 emulated breast cancer comparisons. Nearly half (12/25, 48%) the ovarian cancer cases had likely hereditary *BRCA* mutations. The remaining sporadic ovarian cancers gave the same results as the complete set but with less data. As in breast cancer, ovarian cancer breakpoint distributions clustered around NPC breakpoints, even without a hereditary *BRCA1* or *BRCA2* gene mutation driver.

### Breaks in Lymphomas Associated With EBV Infection Also Matched Breast Cancer and NPC

**Figure 2.**
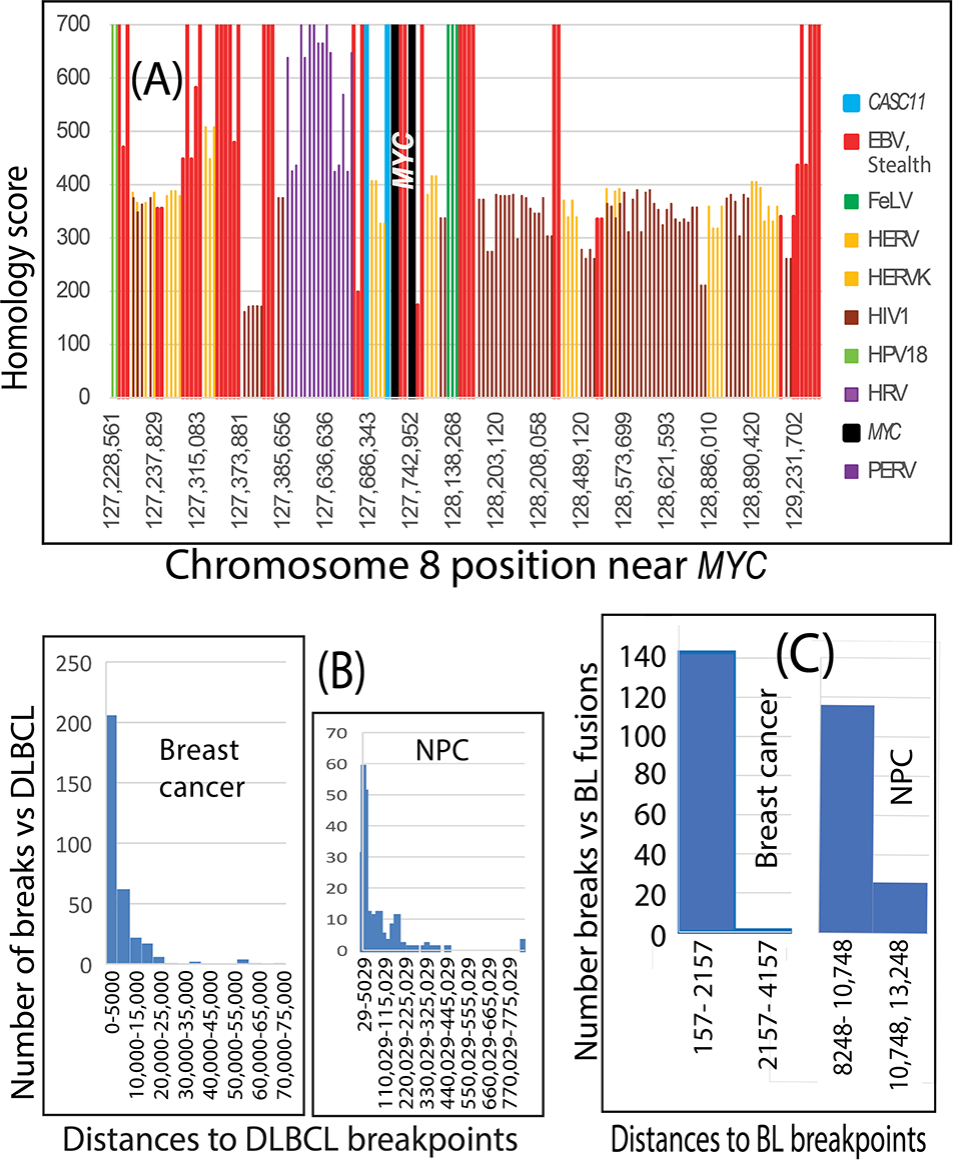
(A) Human DNA around the *MYC* locus on chromosome 8 was filled with virus-like sequences. *CASC11* is an RNA gene that several cancers overexpress. Breast cancer and lymphoma breakpoints were dispersed throughout the *MYC* region and beyond, but NPC breakpoints were less common. (B) On chromosome 8, hundreds of breakpoints in breast cancers and NPCs clustered around breakpoints in data from 88 patients with DLBCL who were likely EBV positive. This agreement highlights multiple similarities among these cancers. (C) EBV drives a subset of BLs, typically with *MYC* translocations and impaired homologous recombination. Based on *MYC* fusion sequences in BL, breast cancer breakpoints on chromosome 8 also clustered around BL breakpoints. BLs from an older dataset [21] had translocation breakpoints in the virus-rich area near the *MYC* locus, agreeing with ≥140 breast cancer breakpoints. *MYC* locus translocations had not been reported in NPCs, but NPC breakpoints still clustered around BL fusion breakpoints, although at greater distances. Four different NPC breakpoints produced over 100 matches to BL translocation breakpoints beginning at about 8250 bp apart. An unpaired, 2-tailed *t* test did not support a statistically significant difference between BL and NPC breakpoints in this area (*P*=.69). BL: Burkitt lymphoma; bp: base pairs; DLBCL: diffuse large B-cell lymphoma; EBV: Epstein-Barr virus; FeLV: feline leukemia virus; HERV: human endogenous retrovirus; HERVK: human endogenous retrovirus K; HIV1: human immunodeficiency virus type 1; HPV18: human papillomavirus 18; HRV: human retrovirus; NPC: nasopharyngeal cancer; PERV: porcine endogenous retrovirus; Stealth: stealth virus 1.

EBV drives lymphomas as well as NPCs. Based on epidemiologic research results, FA-BRCA pathways protect against lymphomas [59,60]. If EBV is genuinely associated with breast cancer breakpoints, then breakpoint positions in EBV-mediated lymphomas should also resemble those of breast and ovarian cancers. Because *MYC* gene rearrangements are characteristic of EBV-associated lymphomas, the first test of this idea was to survey virus-like sequences surrounding the *MYC* gene locus on the human reference genome. Figure 2 shows that *MYC* resides in a literal forest of retrovirus sequences (eg, human immunodeficiency virus type 1 [HIV1], feline leukemia virus, porcine endogenous retrovirus, and human endogenous retrovirus [HERV]) interspersed with EBV-like sequences.

The concentration of virus sequences around *MYC* on chromosome 8 prompted the addition of the EBV-associated lymphoma DLBCL to breakpoint comparisons. As shown in Figure 2, the results revealed that hundreds of breast cancer and NPC breakpoints congregated around breakpoint positions in 88 DLBCLs [22]. This agreement was consistent with other similarities between breast cancers and these EBV-associated cancers, including deficits in FA-BRCA pathway–mediated DNA repair by homologous recombination [61] and NF-κB activation [11,62-64].

EBV is also a proven driver of at least one subset of BLs, typically those with *MYC* translocations. BL subsets can have mutations that impair homologous recombination [65], so results in Figure 2 revealed many breast cancer breakpoint positions near corresponding BL breakpoints. An older dataset from BLs [21] had translocation breakpoints in the virus sequence–rich area near the *MYC* locus, agreeing with about 140 breast cancer breakpoints. Four different NPC breakpoints produced over 100 matches to BL translocation breakpoints, beginning at 8250 bp apart. An unpaired, 2-tailed *t* test did not support a statistically significant difference between BL and NPC breakpoints in this area (*P*=.69).

**Figure 3.**
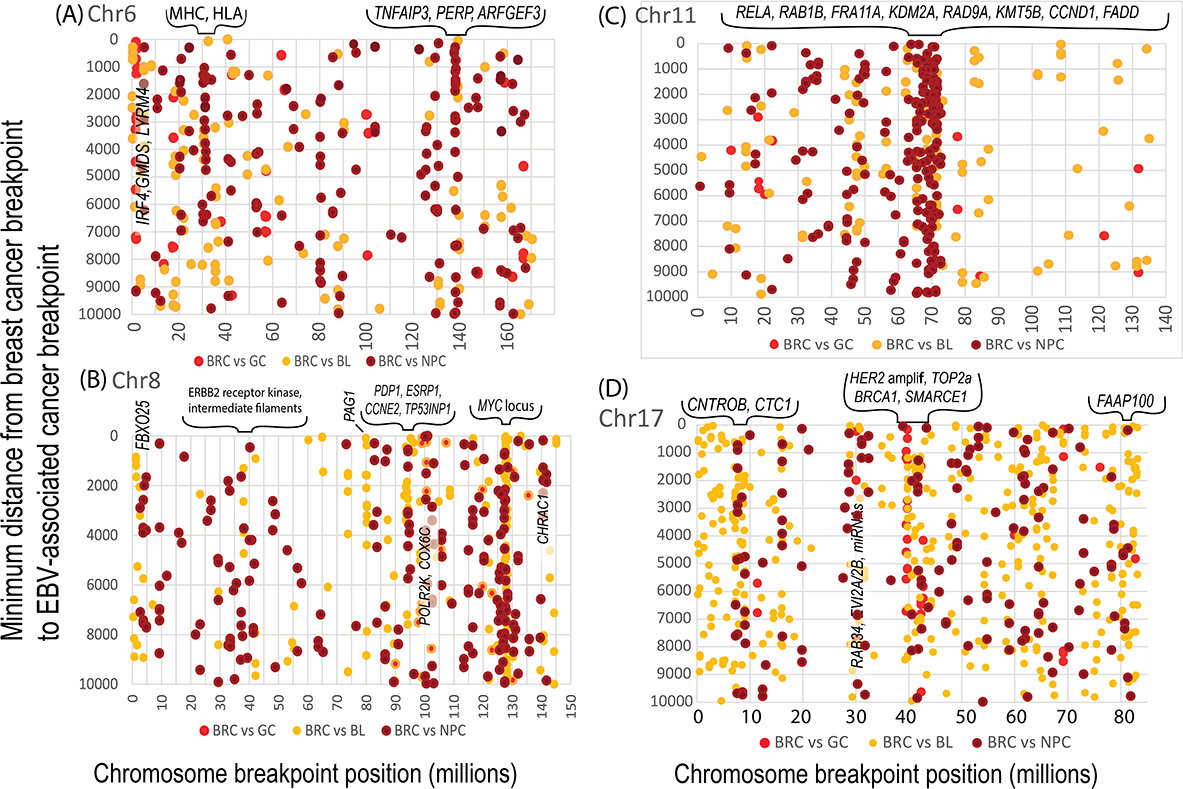
Breakpoints in breast cancers clustered around breakpoints in EBV-positive cancers in 3 different tissues. The EBV-positive cancers comprised 34 GCs, 90 BLs, and 70 NPCs. The clustering of breast cancer breakpoints and EBV-related cancer breakpoints was pronounced on chromosomes (A) 6, (B) 8, (C) 11, and (D) 17. Selected genes around some of the clustered breaks are indicated. Functions of the genes can have profound effects on the human genome and are summarized in Table S1 in Multimedia Appendix 4. BL: Burkitt lymphoma; BRC: breast cancer; Chr: chromosome; EBV: Epstein-Barr virus; GC: gastric cancer; HLA: human leukocyte antigen; MHC: major histocompatibility complex; NPC: nasopharyngeal cancer.

Further tests were conducted to determine whether the functions of genes near clustered breakpoints supported a relationship between breast cancers and EBV-related cancers (GC [41], BL, and NPC). As illustrated in Figure 3, breast cancer breakpoints on chromosomes 6, 8, 11, and 17 aggregated near positions where breakpoints occurred in EBV-associated cancers. Many aggregated breakpoints were in the same areas as genes that control inflammation, antiviral defenses, apoptosis, intermediate filaments, epigenetic and chromatin regulation, estrogen receptor activity, mitotic structures, and mitotic controls (Table S1 in Multimedia Appendix 4). Breast cancer breakpoints that clustered around EBV-associated cancer breakpoints were especially numerous on chromosome 17. One of these clusters marked in Figure 3 included the HER2 amplicon and the topoisomerase 2a gene, with *BRCA1* and *SMARCE1* genes nearby. *SMARCE1* encodes a part of a chromatin regulation complex. Chromosome 17 breakpoints near *CNTROB* and *CTC1* genes connect EBV to centriole and telomere malfunctions during mitosis (Table S1 in Multimedia Appendix 4). Rearrangements near breakpoints may cause over- or underexpression of nearby genes (Table S2 in Multimedia Appendix 4). Many additional correlations were also likely revealed in Figure 3 but were not investigated further.

Results in this section show that breast cancer breakpoints clustered around breakpoints in additional EBV-associated cancers, where they affect critical functions needed to prevent breast cancer. Once these functions are compromised, cancer can occur without the continuing presence of EBV.

### Genes at the Most Frequent EBV-Tethering Sites Clustered Around Breast Cancer Breakpoints

**Figure 4.**
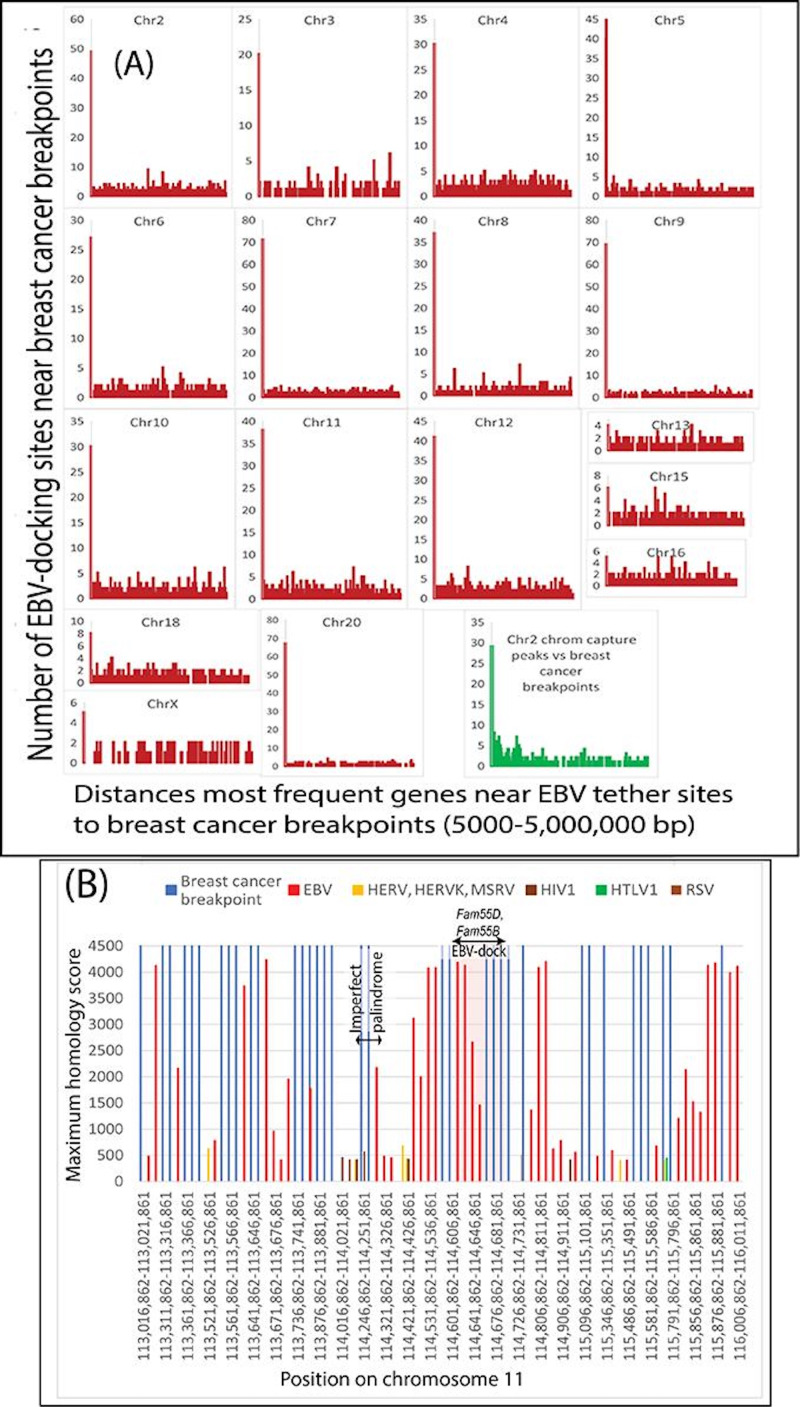
Relationships of EBV-docking sites to breast cancer breakpoints. (A) Breast cancer breakpoints clustered around the top 10% most frequently found genes near EBV-tethering sites in BL cells. Some of the best information on EBV-docking sites comes from 4C-chromatin capture experiments in EBV-positive BL cells [47]. The largest number of breast cancer breakpoints on most chromosomes clustered around the genes nearest EBV-tethering sites. BL cells providing the data had up to 1569 EBV-docking sites distributed over all chromosomes [47]. EBV-docking sites on chromosome 11 near the *LUZP2* and *FAT3* genes in BL cells were millions of bp from the 18-bp imperfect palindrome interval. Graphical estimation of virus-tethering sites on chromosome 2 (green) from these EBV-positive cells also agreed with breast cancer breakpoints. (B) Independent evidence relating breast cancer chromosome breakpoints to EBV-docking sites. Maximum homology to human DNA for all viruses (y-axis) is plotted around known EBV genome anchor sites on chromosome 11 near the *FAM55D* and *FAM55B* gene coordinates. A posited imperfect palindrome sequence [66] as an EBV-docking site was more distant from the *FAM55* genes. BL: Burkitt lymphoma; bp: base pairs; Chr: chromosome; chrom: chromatin; EBV: Epstein-Barr virus; HERV: human endogenous retrovirus; HERVK; human endogenous retrovirus K; HIV1: human immunodeficiency virus type 1; HTLV1: human T-cell lymphotropic virus type 1; MSRV: multiple sclerosis retrovirus; RSV: respiratory syncytial virus.

In preceding sections, breast and ovarian cancer breakpoints were found to distribute most frequently near characteristic sets of breakpoints associated with EBV-related cancers. The virus first attaches its EBNA1 protein to human DNA in the nucleus. Then, circular EBV episomes dock to this attached EBNA1 anchor. To test whether the initial EBNA1 attachment sites were related to breast cancer chromosome breakpoints, breast cancer breakpoints were compared to genes near EBV-docking sites. EBV-positive BL cells providing the data had up to 1569 EBV-docking sites on all chromosomes identified by 4C-chromatin capture experiments [47]. As shown in Figure 4A, the largest numbers of breast cancer breakpoints on most chromosomes clustered around the genes [47] nearest to genes at EBV-docking sites. In support of these comparisons, graphical estimation of virus-tethering sites on chromosome 2 from chromatin capture data for these EBV-positive cells also agreed with breast cancer breakpoints (Figure 4A). In an unrelated study [48], EBV-docking sites on chromosome 11 near known EBV anchor sites at the *FAM-D* and *FAM-B* genes were found near groups of breast cancer breakpoints, but imperfect palindrome sequences [66] were more distant (Figure 4B). This finding independently supports the idea that EBV-docking sites are near breast cancer breakpoints. Results in this section raise the possibility that EBV directly contributes to breast cancer chromosome breakpoints and fragmentation.

### Breakpoints Occurred Near Human Sequences That Resemble Viruses in All Breast Cancers Tested

**Figure 5.**
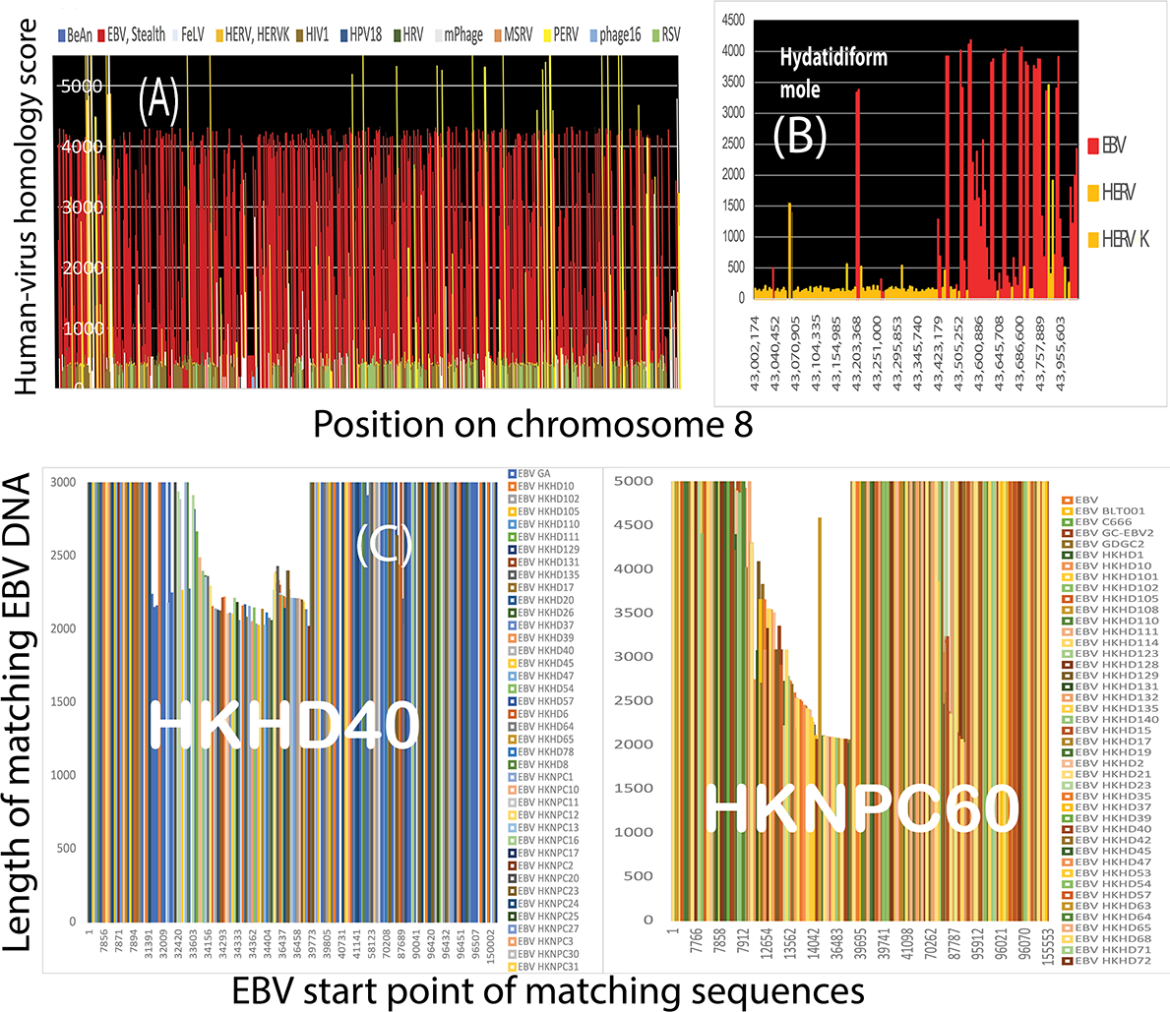
(A) All viral homologies on the entire lengths of chromosome 8 (a total of 145,138,636 bp) are shown in 200k-bp increments. Maximum homology scores over 4000 for human DNA versus herpes viral DNA were abundant. The 4000 score corresponds to 97% human-virus identity over nearly 2500 bp, with *E* (“expect”) values (essentially *P* values) effectively equal to 0. The EBV tumor variants, HKNPC60 and HKHD40, were nearly identical to human breast cancer DNA at many positions throughout chromosome 8. (B) It is unlikely that homologies to EBV sequences occurred because the human reference genome was contaminated with EBV episomes. Homozygous hydatidiform mole cells that had lost the paternal chromosomes after fertilization still had strong homology to EBV sequences, such as HKHD40 and HKNPC60 variants. (C) EBV variants HKHD40 and HKNPC60 are typical of hundreds of other EBV variants. Hundreds of human gamma herpesvirus 4 variants are almost identical to HKHD40 and HKNPC60 over at least 2000 bp. The matching sets of viruses included many high-risk herpesvirus isolates from NPCs [67]. BeAn: BeAn 58058 virus; bp: base pairs; EBV: Epstein-Barr virus; FeLV: feline leukemia virus; HERV: human endogenous retrovirus; HERVK: human endogenous retrovirus K; HIV1: human immunodeficiency virus type 1; HPV18: human papillomavirus 18; HRV: human retrovirus; mPhage: mycolicibacterium phage J1; MSRV: multiple sclerosis retrovirus; NPC: nasopharyngeal cancer; PERV: porcine endogenous retrovirus; RSV: respiratory syncytial virus; Stealth: stealth virus 1.

To further test whether EBV itself has some role in breaking chromosomes or altering their structures, human chromosomes were compared to all known viruses. As shown in Figure 5A, the results showed that nearly every breast cancer likely had undergone breakages near EBV-like sequences. Chromosome 8 alone had 59,566 significant (>200) viral homology scores. Based on data from 128 patients with breast cancers and 43,491 unique breakpoints, breakpoints in 123 (96.1%) out of 128 breast cancers were within 10,000 bp of a virus sequence. In 106 patients, the virus was an EBV tumor variant (HKHD40 or HKNPC60) with 3086 matching human sequences. According to the Fisher exact test, chromosome 8 breakpoints and EBV variant sequence matches were not independent (*P*<.001).

Many areas on other chromosomes also had 97% human-virus identity over nearly 2500 bp. It is implausible that this much similarity comes from EBV DNA being carried over into the human reference genome. Viral homology occurred with only a small, select portion of viral DNA [68]. Viral homologies were determined for a human genome in a homozygous karyotype, haploid cell line (46,XX) hydatidiform mole derived only from the paternal chromosomes in an X-bearing sperm cell after fertilization [69]. Results still showed extensive homology between the mole and EBV variants HKHD40 and HKNPC60 (Figure 5B).

HKHD40 and HKNPC60 variant sequences kept appearing in comparisons to human sequences, so these variants were tested against other herpesviruses to determine whether they were unusual. Hundreds of human gamma herpesvirus 4 variants were almost identical to HKHD40 and HKNPC60 over at least 2000 bp (Figure 5C). The matching sets of viruses included many high-risk herpesvirus isolates from NPCs [67]. Based on this information, HKHD40 and HKNPC60 strongly resembled other herpesvirus isolates, including many that confer high risks for NPC [10]. These results show that humans have interacted extensively with EBV; the results are not due to EBV impurities in the human reference genome, and the human genome has had close relationships with oncogenic EBV forms.

### Evidence of Past EBV Infection

The evidence thus far supports a central hypothesis that EBV disables tumor suppressor mechanisms in breast cancer and can then disappear. This absence of viral particles is a significant experimental obstacle to testing this hypothesis. Unlike retroviruses, EBV and its variants do not have integrase enzymes, so EBV has no conventional way to insert itself into the human genome. EBV rarely integrates, with only one or two copies in BL cell lines [70].

BLAST analysis found about 65,000 areas of strong homology (*E*<1×10^–10^) between the human reference genome and EBV. Because 65,000 is far more than realistic EBV integration events, it suggested the possibility that some EBV sequences were fragments created by a human version of the bacterial CRISPR (clustered regularly interspaced short palindromic repeats) system. As shown previously in Figure 3, breast cancers have breakpoints that cluster around breakpoints in EBV-associated cancers and involve MHC genes.

MHC genes are encoded on chromosome 6p21.3 in a region that becomes a candidate for such a human CRISPR version. Variants of human leukocyte antigens (HLAs) in the MHC are strong risk factors for NPC infections [71] because HLAs are required to break down and display fragments of some antigens to the immune system. A total of 13 breast cancers listed on the COSMIC website had a deletion near this HLA region. About 23% of breast cancers had mutations directly affecting HLA class I or II genes. Many more breast cancers had indirect connections because they had damage to multiple genes that interact with HLAs or were otherwise essential for immunity. The MHC region also holds *NFKB1L1*, a negative regulator of the NPC overexpressed gene hallmark, NF-κB. The 139 breast cancers from high-risk women had 284 breakpoints at chromosome 6p21.3. Breakpoints in the 70 NPC cancers also clustered there, with 40 breakpoints within the 27,865,296-34,017,013 segment on chromosome 6. Variability in the inactivation of MHC genes reflects the extreme diversity of this region.

**Figure 6.**
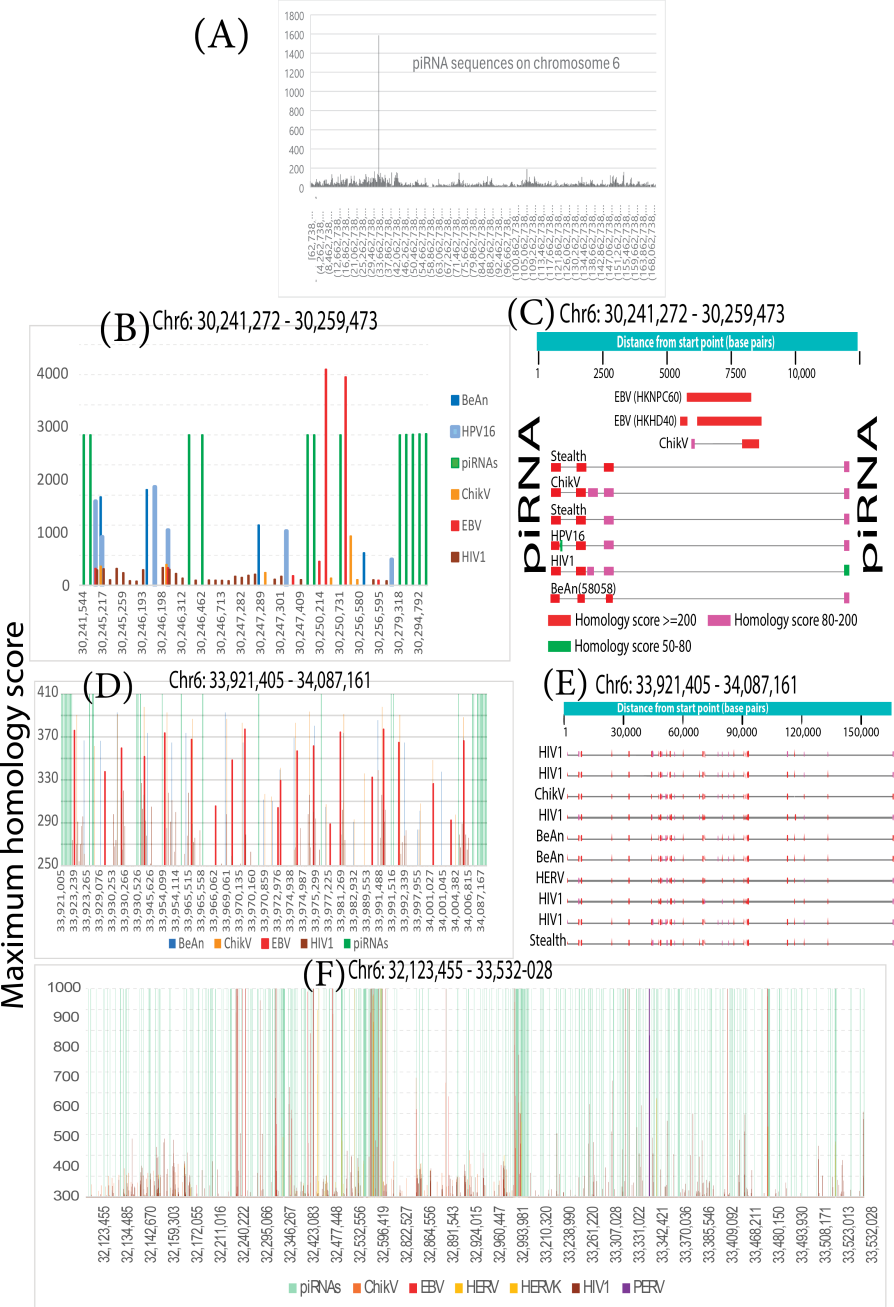
The human genome organizes piRNA sequences into clusters near the MHC region of chromosome 6 (6p21.3 at ~29.7-33.3 megabases), with hundreds of piRNAs nearby. (A) The levels of various piRNAs varied by more than 1000-fold, but the most abundant piRNAs were the only ones present in every cell. These abundant sequences drive the inactivation of foreign DNA. Rare piRNAs do not function in every cell but can potentially adapt to new genome invaders. (B-F) Arbitrarily selected areas of the chromosome region where piRNAs are most abundant. piRNAs were assigned sufficient homology scores to mark their positions relative to positions with homology to viruses. (C and E) Remnants of both exogenous and endogenous virus types were homologous to the same human sequence, and both types were sandwiched between piRNA sequences, sometimes right next to each other. Most sandwiches were at a regular interval or a multiple of a regular interval. The same human DNA interval has homology to endogenous transposons (HERV) and exogenous viral sequences (ChikV, HIV1, Stealth, BeAn, and HPV16). The piRNA system can store the same piece of DNA to protect DNA against these different viruses. BeAn: BeAn 58058 virus; ChikV: chikungunya virus; Chr: chromosome; EBV: Epstein-Barr virus; HERV: human endogenous retrovirus; HERVK; human endogenous retrovirus K; HIV1: human immunodeficiency virus type 1; HPV16: human papillomavirus 16; MHC: major histocompatibility complex; PERV: porcine endogenous retrovirus; piRNA: Piwi-interacting RNA; Stealth: stealth virus 1.

In general, the bacterial CRISPR/Cas system loosely resembles the human piRNA system, so the distribution of piRNAs was graphed. As shown in Figure 6A, hundreds of piRNA sequences cluster near the MHC region (at ~29.7‐33.3 megabases). The piRNA system is known to inactivate virus-derived transposons (related to HERVs) by methylating or cleaving them. The distribution of piRNA fragments was then compared to the distribution of viral DNA fragments in the MHC region of chromosome 6. Figure 6B-F reveals striking similarities in how remnants of exogenous and endogenous viruses distribute relative to piRNAs. Remnants of both virus types were homologous to the same human sequence, and both types were interspaced between piRNA sequences, sometimes right next to each other. Most of these sandwiches were at a regular interval or a multiple of a regular interval.

This interspaced arrangement looked so much like CRISPR that it raised the question of whether piRNA defense mechanisms have inactivated some EBV variants in addition to their canonical role with endogenous viruses. Long stretches of endogenous transposon-like DNA sequences routinely matched exogenous viruses. As shown in Figure 6C and E, the same human DNA interval had homology both to endogenous transposons (HERV) and exogenous viral sequences (EBV variants, stealth virus 1, chikungunya virus, BeAn 58058 virus, human papillomavirus [HPV] 16, HIV1, and HERV). This result shows that the piRNA system can store the same piece of DNA to protect DNA against these different viruses.

**Figure 7.**
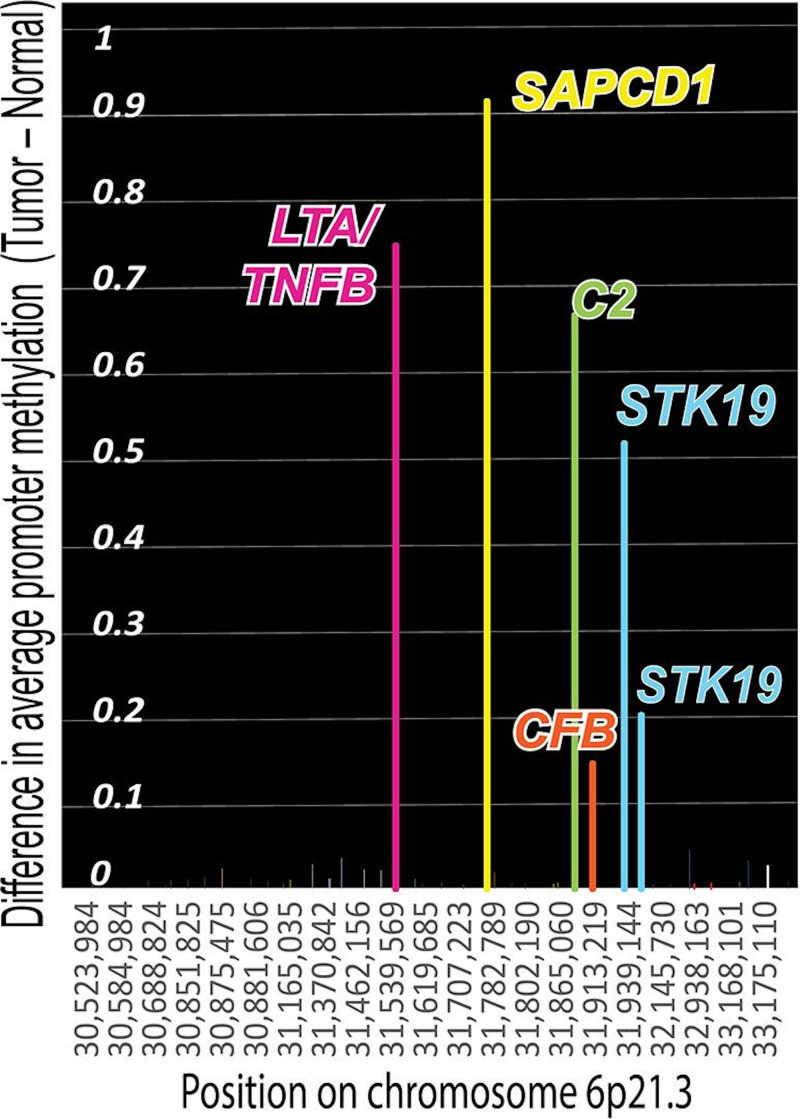
Chromosome 6p21.3 contains an EBV infection signature [72]. Using existing methylation data [34], the marker was examined in 1538 breast cancers. Promoter methylation in this marker region differed significantly from normal controls. Hypermethylation occurs on *STK19*, an MHC class III region gene [73] for RNA surveillance [74]. Hypermethylation also inhibited *LTA/TNFB*, a gene for preventing tumors [75], and *C2*, which encodes antigen-antibody complex responses. Polymorphisms in *HLA-DMB* antigen and *SAPCD1*, another class III MHC gene [76], at chromosome 6p21.3 have links to Kaposi sarcoma [77]. HHV8 is a Kaposi sarcoma virus closely related to EBV. EBV: Epstein-Barr virus; HHV8: human herpesvirus 8; HLA: human leukocyte antigen; MHC: major histocompatibility complex.

Chromosome 6p21.3 also contains an EBV infection marker [72]. The marker was examined in 1538 breast cancers using existing methylation data [34]. As indicated in Figure 7, promoter methylation differed significantly from normal controls in the segment shown (30,523,984‐33,216,811 on chromosome 6). Hypermethylation occurred on *STK19*, a MHC class III gene for RNA surveillance [73,74]. Hypermethylation also occurred on a gene for preventing tumors (*TNFB*) [75] and a gene for responding to antigen-antibody complexes (*C2*). Polymorphisms in *HLA-DMB* antigen and *SAPCD1*, another class III MHC gene [76], at chromosome 6p21.3 had links to Kaposi sarcoma [77]. Human herpesvirus 8 (Kaposi sarcoma virus) is a Kaposi sarcoma driver and is closely related to EBV.

These results reveal that EBV has been attacking human DNA during evolution. There is a piRNA defense mechanism for human DNA near critical immune system genes, but both EBV-associated cancers and breast cancers inactivate some of the genes that guard piRNA defenses. The histocompatibility antigen gene region of chromosome 6 can be extensively fragmented in EBV-associated and breast cancers. MHC genes have the largest number of polymorphic forms in the human genome. This variation creates differences in viral susceptibility and inactivation. Even though most people are infected, not everyone will get an EBV-related disease or cancer.

### Viral Sequences in Human Genomes as Hypermutation and Rearrangement Sites in Breast Cancers

**Figure 8.**
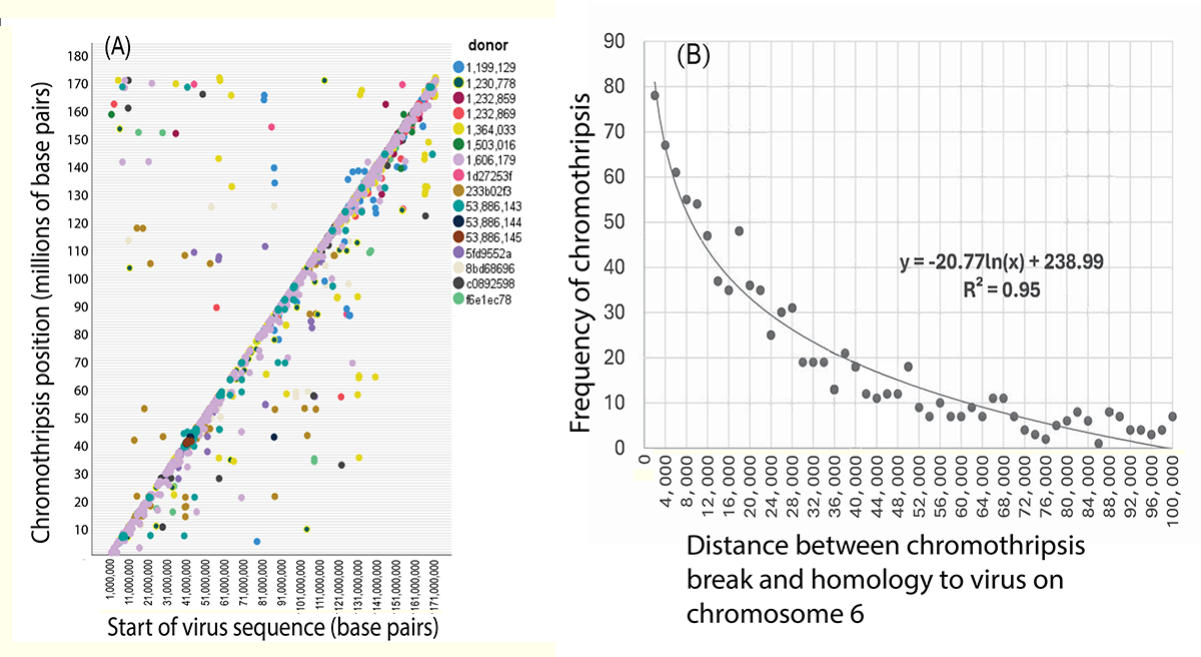
Repetitive copies of virus sequences may confuse compromised DNA repairs and contribute to hypermutation clusters and rearrangements. (A) High-confidence positions where chromosome 6 shatters in 16 breast cancer genomes [35] were plotted against start points of viral sequence homologies. EBV or other viruses then cause groups of rearrangements and hypermutation clusters (chromothripsis). A total of 1090 genome coordinates described fragments with copy number ≥3. These coordinates were unlikely to be completely random since they did not follow a normal distribution (*P*<.001). Genome coordinates on chromosome 6 matching virus sequences were strongly correlated by simple linear regression analysis (*R*^2^=0.93). (B) As you move further from chromothripsis breakpoints, the frequency of breast cancer homology to viruses decreases, according to the equation shown. The constant in the equation suggests a baseline level of breaks.

The next question was whether EBV or other virus-like sequences in the human genome cause multiple rearrangements and clustered hypermutations (chromothripsis). As shown in Figure 8A, many positions on chromosome 6 where chromothripsis occurs [35] congregated around virus sequence start positions. A total of 1090 genome coordinates described chromothripsis fragments with copy number ≥3. These coordinates were unlikely to be random since they did not follow a normal distribution (*P*<.001). By simple linear regression analysis (*R*^2^=0.93), many viral sequence coordinates strongly correlated with chromothripsis positions. Figure 8B shows that as you move further away from a chromothripsis breakpoint, the frequency of breast cancer homology (score>500) to viruses decreases. This result implicates viral sequences as preferred sites where breast cancer chromosomes begin to fall apart. The equation shown mathematically describes the relationship between chromothripsis frequency and distance from viral sequences, and the constant in the equation suggests a baseline level of breakpoints.

These results suggest that homologous virus sequences at multiple positions could confuse DNA repairs already compromised by EBV in breast cancer and contribute to chromothripsis and clustered rearrangements.

### EBV and Metastasis

The last question was whether EBV contributes to breast cancer metastasis. According to Yates et al [29], relapsed and metastatic breast cancer tumors keep their tumor-driver gene mutations and continue acquiring new ones. Late mutations in JAK-STAT and SWI-SNF signaling pathways drive established breast cancers into metastasis.

**Figure 9.**
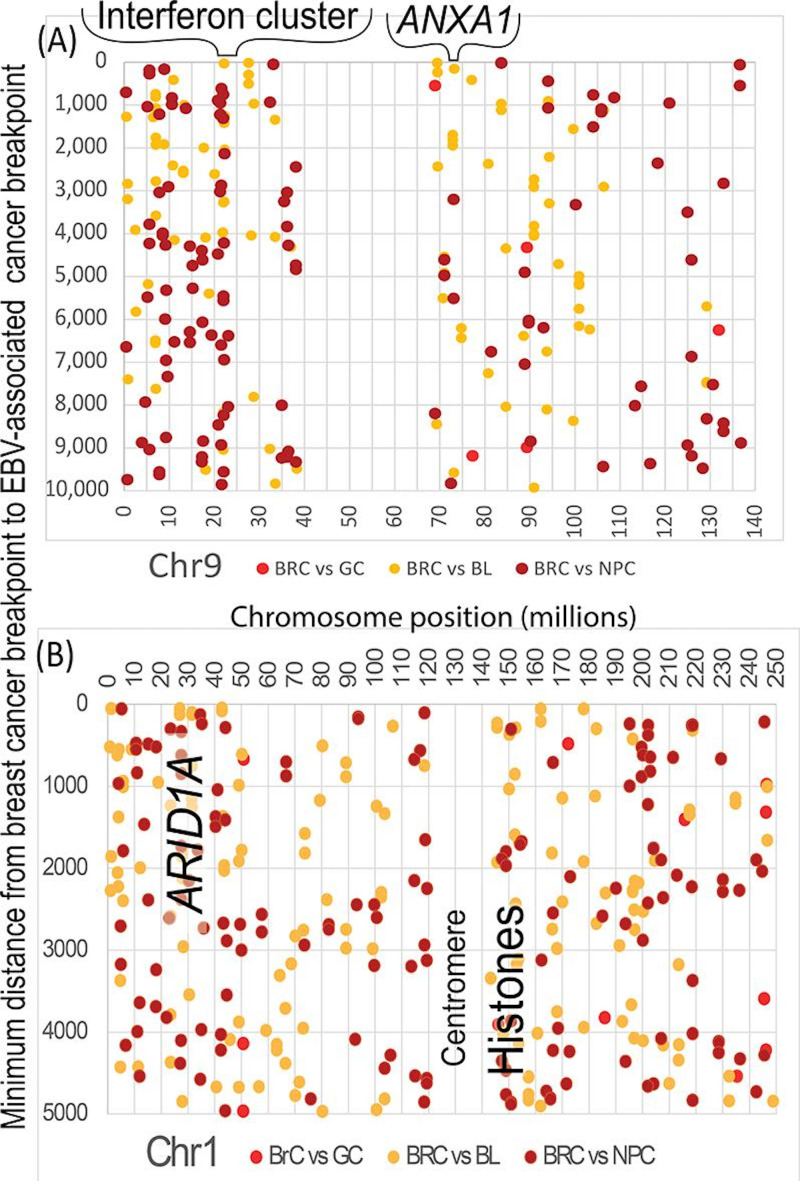
Damage to JAK-STAT and SWI-SNF signals pushes breast cancer into metastasis [29]. EBV interferes with these signaling pathways to facilitate viral replication. (A) Breakpoints in breast cancers on chromosome 9 facilitated viral replication and blocked sources of JAK-STAT signaling, including a large cluster of interferon genes on chromosome 9. Breast cancers can disable SWI-SNF by targeting *ARID* genes. (B) *ARID1A* was encoded on chromosome 1 near a hot spot where multiple breast cancer breakpoints approximately aligned with breakage points in EBV-associated cancers. Another site at about 150,000,000 bp had a histone-rich region nearby. SWI-SNF affects histones, which also profoundly affects metastasis [78]. The GRCh38 genome version does not include centromere sequences due to technical limitations. *ANXA1*: Annexin A1; BL: Burkitt lymphoma; bp: base pairs; BRC: breast cancer; Chr: chromosome; EBV: Epstein-Barr virus; GC: gastric cancer; NPC: nasopharyngeal cancer; SWI-SNF: switch/sucrose non-fermentable.

NPC often loses type-1 interferon genes (*IFNA1*, *IFNA2*, *IFNA8*, and *IFNE*) and nearby *MTAP* (32%-34% [11]) by homozygous deletions at chromosome 9p21.3. Interferons initiate canonical JAK-STAT signaling by binding to cell surface receptors that then activate internal Janus kinases (JAKs). The activated JAKs phosphorylate cytoplasmic STAT (signal transducer and activator of transcription) proteins, which travel to the cell nucleus to activate interferon-responsive genes. The percentages of breast cancers on the COSMIC website with mutations in a “JAK” or “STAT” isoform or transcript variant were calculated: 7.8% had a JAK mutation and 36.7% had a STAT mutation. Deletions of interferon genes in NPC also facilitate viral replication and block interferon from activating JAK-STAT signaling. Breast cancers (Multimedia Appendix 2) have 65 breakpoints strictly within this interferon-*MTAP* region (21,579,478‐20,503,534 on chromosome 9), not counting longer fragments that include the interval. As shown in Figure 9, breast cancer breakpoints align well with EBV-associated cancer breakpoints near the large cluster of interferon genes on chromosome 9.

Mutations in EBV-associated cancers show that Yates metastasis driver gene damage accompanies EBV infection. SWI-SNF (switch/sucrose non-fermentable) is a complex that repositions nucleosomes and supports genome stability [79]. SWI-SNF addresses obstacles to replication sensed by the FA-BRCA pathway [79,80]. Referring back to Figure 3, clustered breast cancer breakpoints on chromosome 17 around EBV breakpoints affect the SWI-SNF component *SMARCE1*. In addition, breast cancers can disable SWI-SNF by targeting *ARID* genes [29]. *ARID1A* is a COSMIC top-20 most frequently mutated gene in breast cancer. Like breast cancer, NPC has multiple recurrent aberrations in *ARID1A* genes. As shown in Figure 9, *ARID1A* lies near a hot spot where multiple breast cancer breakpoints approximately aligned with breakage points in EBV-associated cancers. The loss of *ARID1A* activates Annexin A1, which aligned closely with a region targeted by EBV-associated cancers on chromosome 9. A chromosome-1 site at about 150 million bp had a nearby histone-rich gene region. Histones are chromatin structures that SWI-SNF dynamically remodels to regulate access to genetic information. Histones can profoundly affect metastasis [78]. Figure 9 also reveals many additional alignments between breakpoints in breast and EBV-associated cancers that were not investigated further.

NPC often inactivates SWI-SNF components *BAP1* and *PBRM1* within a frequently damaged 3p21.3 gene cluster [11] at 52,400,000‐53,000,000 on chromosome 3. Analyses of breast cancers found 18 breakpoints within this short interval. DLBCL, another EBV-linked cancer, also had recurrent alterations in components of SWI-SNF complexes [81].

The Warburg effect (oxidative glycolysis) [68] favors metastasis. The Warburg effect occurs in NPC because pyruvate dehydrogenase *(PDHB)* genes on chromosome 3p are deleted or rearranged in almost all cases. Similar changes to chromosome 3p were found in breast cancers, which also undergo the Warburg effect [68]. This Warburg metabolic switch favors metastasis because it mitigates oxidative stress on cancer cells. Large amounts of lactate accumulate in the absence of *PDHB* to acidify the tumor microenvironment and interfere with the destruction of metastatic cells [82].

This section’s results show that EBV may push breast cancer into metastasis by interfering with JAK-STAT and SWI-SNF signaling pathways to facilitate viral replication while making the microenvironment more favorable to tumor growth.

### Alternative Explanations for Breast Cancer Breakpoints That Do Not Involve EBV Variants

#### Subgroups

To determine whether breakpoint similarities in viral and breast cancers depended on specific subgroups, relationships to NPC were compared in triple-negative and HER2-positive breast cancers (20 and 22 patients, respectively). Triple-negative breast cancers are likely to be *BRCA1* mutation positive [83], while HER2 amplification is uncommon in *BRCA1* and *BRCA2* mutation carriers [84]. Although subgroup differences are noticeable, results still show that both subgroups had breakpoints on all chromosomes related to NPC (Figure S1A in Multimedia Appendix 5).

#### Tumor-Infiltrating Lymphocytes

Tumor-infiltrating lymphocytes (TILs) are biomarkers for predicting breast cancer prognosis [85,86]. To test whether TILs cause chromosome breaks, breakpoint numbers in 16 breast cancers with severe lymphocyte infiltration were compared to 17 breast cancers with nil lymphocyte infiltration. The 2-tailed Student *t* test could not reject the null hypothesis that the numbers of breakpoints were statistically identical (*P*=.70; Figure S1B in Multimedia Appendix 5). This result does not rule out differences in prognosis due to differences in lymphocyte infiltration.

#### Retroviruses

Retrovirus contributions to structural variations were estimated using data from cancer in 38 different tissues [87]. Retrotransposons make relatively modest contributions to breast cancer compared to, say, esophageal or oral (gums) cancer (Multimedia Appendix 5). EBV can transactivate endogenous retroviruses [11,87,88]. DNA near some breast cancer breakpoints resembles porcine endogenous retrovirus, HERV, and HIV1 (eg, Figure 5). The human genome also contains DNA matching the retrovirus mouse mammary tumor virus [89,90] at 23 sites that give BLAST homology scores>200. HPV variants are DNA viruses that are also implicated in breast cancer. HPVs were not assessed further, but they occasionally matched DNA near breast cancer breakpoints.

#### Common Fragile Sites

Common fragile sites are site-specific breaks seen on metaphase chromosomes after inhibiting DNA synthesis via DNA polymerase inhibitors. Some common fragile sites [54] aligned with breast cancer breaks on chromosome 1, but breakpoints on most other chromosomes were incompatible. Chromosomes 8, 9, 11‐15, 17-19, 21, and 22 do not have common fragile sites but still have many breast cancer breaks [91]. However, the human genome has over 13 million palindromes that are ≤40 bp [92]. The generation of rare fragile sites by palindromes or their attraction to EBV cannot be excluded.

#### Imperfect Palindrome Repeats

An alternative explanation for EBV-related carcinogenesis involves the docking of EBNA1 virus-tethering protein at imperfect palindromes [93] tandemly repeated on chromosome 11. The docked EBNA1 binds EBV circular episomes, and chromosome 11 breaks initiate malignancy. To test this explanation, existing literature data were first compared to the specific human EBNA1-binding site [48,66,94]. The results (Table S2 in Multimedia Appendix 4) are incompatible with a single host sequence binding EBNA1.

BLAST analysis showed that matches to the imperfect palindrome were likely due to pure chance with *E* values between 16 and 964 for 4352 matches, from 12 to 18 bp. Chromosome 11 had only 197 of these 4352 matches, and none were near the palindromic region. The prototype DNA palindrome (Table S2 in Multimedia Appendix 4, line 2) produced 7074 matches with *E* values ranging from 0.25 to 964. Further BLAST analyses of the slightly different docking sequence in EBNA1-DNA crystals (Table S3 in Multimedia Appendix 4, line 1) against other genome assemblies [95] revealed matches on chromosomes 2, 19, 4, and 12. Various isolates of HIVs had 52 matching sequences.

In 94 BL samples from patients who were EBV positive, breakpoints concentrated within chromosomes 2, 8, 13, 14, and 22 (Figure S1D in Multimedia Appendix 5). Chromosome 14 contained 610 breakpoints (*IgVH* regions), and chromosome 2 (*IgVK* regions) contained 522 breakpoints. EBV hijacks activation-induced cytidine deaminase, a mutagenic enzyme that generates antibody gene variants in response to myriad antigens. In the 94 EBV-positive BL cases, the palindromic locus was nearly 100 million bp away from the principal breakpoint coordinates (Figure S1E in Multimedia Appendix 5). Only 19 (20%) of the 94 patients who were EBV positive [96] had breakpoints anywhere on chromosome 11. The palindromic locus was also not involved in diverse cancers from 8227 patients [97] (Multimedia Appendix 5).

The results in this section show that alternative explanations that invoke subgroups, TILs, retroviruses, or a specific palindromic repeat locus are incompatible with the associations between EBV-associated and breast cancers .

## Discussion

### Principal Findings

**Figure 10.**
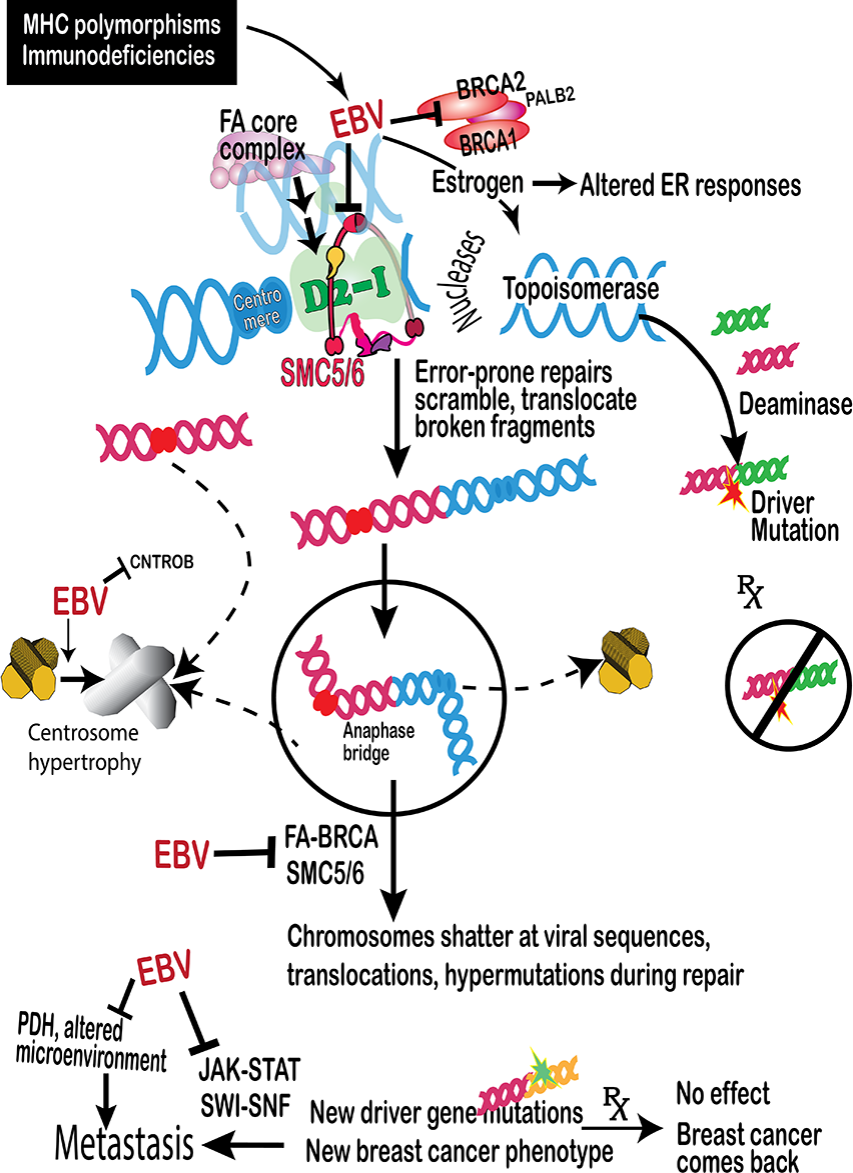
Model proposed to explain the results. EBV causes serious disease in only some people due to MHC variants and other damage to the immune system. Viral nucleases are one source of chromosome breaks. EBV causes inappropriate expression of estrogen and transcription targets of occupied estrogen receptors. Transcription induced by artificially high estrogen levels then induces topoisomerase-mediated DNA breaks. EBV-mediated deregulation of estrogen production, topoisomerase activity, and deaminase activation then collaborate to cause chromosome breaks and drive translocations [68]. EBV-associated cancers share additional genome deficits with breast cancers, which interfere with restoring the genome from DNA crosslinks and DNA double-strand breaks. If crosslinks and DNA breaks persist during cell division, they also cause chromosome rearrangements and cancer. The cancer safeguards targeted by EBV extend to the *BRCA* pathway, FA proteins, an SMC5/6 scaffold, JAK-STAT signaling, and the SWI-SNF chromatin remodeling complex. EBV: Epstein-Barr virus; ER: estrogen receptor; FA: Fanconi anemia; MHC: major histocompatibility complex; PDH: pyruvate dehydrogenase; SWI-SNF: switch/sucrose non-fermentable.

This study finds that EBV contributes to breast cancer by disabling safeguards against tumors. Cancer then occurs because the safeguards remain disabled even if the virus is cleared. Multiple independent analyses identified residual genetic and epigenetic damage in cancer genomes and formed the basis of the model in Figure 10. Breakpoints in breast cancers in high-risk women, sporadic breast cancers, and even ovarian cancers cluster around breakpoints in known EBV-related cancers, including NPC, BL, DLBCL, and GC. Some genes clustered near breakpoints in these diverse EBV-associated cancers are critical to preventing breast cancers. Some breast cancer breakpoints are near genes at EBV-docking sites. Varying numbers of DNA breaks occur within the highly polymorphic forms of MHC region genes on chromosome 6. This damage adds to susceptible polymorphisms and immunodeficiencies to help explain why not everyone develops EBV-related cancers. Near the MHC region on chromosome 6, piRNA sequences are regularly interspaced between viral DNA sequences. The sandwiched arrangements are presumptive evidence of past infection and probably represent a DNA defense mechanism. These defenses fail when chromosome 6 breaks apart near start points of the large number of repetitive viral sequences in the human genome. The viral sequences confuse repairs already damaged by EBV, and bursts of mutation occur where scrambled fragments ligate. EBV disables the most reliable restoration of broken chromosomes back to their native forms, so repairs form structures with multiple centromeres. These structures undergo additional rounds of fragmentation during cell division. The process continually forms new cancer driver mutations and allows cancer to come back after successful therapy (Figure 10). An EBV methylation signature on chromosome 6 was far more abundant in 1538 breast cancers than in normal controls. Finally, EBV facilitates its own replication by damaging JAK-STAT and SWI-SNF signaling pathways, which pushes breast cancer into metastasis, while virus-associated changes on chromosome 3p interfere with the destruction of metastatic cells. Models [8,98] of EBV-infected human mammary cell cultures transplanted into immunosuppressed mice and EBV loss from NPC cells are consistent with these results.

The study herein has current and future clinical implications in addressing cancers and chronic diseases. An early childhood vaccine against EBV may reduce the incidence of breast cancer on a global scale. If this vaccine even approaches the effectiveness of the HPV vaccine for cervical cancer, then the reduction of breast cancer incidence would be substantial. In breast cancer cases where active infection can be demonstrated, immunotherapy or antivirals can be considered. The results also heighten concern about hidden dangers from viral infections. EBV infection leaves behind persistent genome abnormalities (“long EBV”) linked to breast cancer. Not everyone develops an EBV-related cancer even though almost everyone is infected, suggesting risk assessment should include MHC polymorphisms. MHC genes have abundant connections to both EBV infection [99] and breast cancer [100-102].

The strategy of using bioinformatics to identify markers of “long EBV” may well work for other cancers, multiple sclerosis [103], and other chronic diseases that are currently unexplained. Testing for persistent viral damage in genomes from biopsies is a new method for screening for breast cancer risk. The results may inform further prevention and treatment decisions. Cancer drug therapy has focused on finding and destroying cancer-driver gene products. The drugs are initially effective, sometimes for long periods, but then stop working. The cycles represented in Figure 10 are an occult, underlying process that can now be evaluated. Cancer treatment generates new clones that do not exist in the original population [104]. The underlying genome damage and EBV scars continually produce new cancer-driver mutations. Some antigens targeted by successful therapy for hematologic malignancies [105], such as DLBCL, may also be effective for breast cancers. The idea that breast cancers and hematologic malignancies can have similar breakpoints and translocation fusions suggests that there may be many more susceptible targets and that there are options to overcome resistance or tolerance [106]. The findings may further stimulate research into other EBV-associated diseases and cancers, leading to better and broader understanding.

Estrogen has been thought to generate the initial chromosome breakpoints leading to translocations in human breast cancer. However, young boys with BL do not produce estrogen from ovaries, yet Figure 3 shows that their malignant B-cells have many breakpoints [68,107] that approximately match breast cancer breakpoints. Normally, aromatase catalyzes the rate-limiting step in estrogen production [108], and aromatase acting on androgens is the primary source of most estrogens in breast tissue [109]. EBV-infected cells lose control of aromatase activity [108]. An EBV-mediated increase in aromatase activity explains why locations of breakpoints (Multimedia Appendix 5) are relatively independent of estrogen receptor status in breast cancer [68] and resemble locations in lymphoid cells (Figures14 and 9). Transcription in response to artificially high estrogen levels created by EBV then induces topoisomerase-mediated DNA breaks. Double-strand break repair genes remove topoisomerase from these complexes, but damage to this process leaves pathological enzyme complexes still bound at a DNA breakpoint [110-112]. As shown in Figure 3, topoisomerase itself may be damaged. In either case, EBV-mediated deregulation of estrogen production, topoisomerase activity, and deaminases then collaborate to cause chromosome breaks and drive breast cancer.

Breast cancer chromosome breakpoints cluster around genes near EBV-binding sites (Figure 4), further suggesting that EBV participates in causing the breaks. The breaks lead to pathogenic chromosome rearrangements because EBV-induced damage forces restoration into error-prone methods by suppressing FA-BRCA pathway intermediates [14,15]. Repairs using the FA-BRCA pathway [113] need chromatin access, which requires the SMC5/6 cohesin complex [114,115]. In one scenario shown in Figure 10, SMC5/6 interacts with a crucial pathway intermediate, the FANCD2-FANCI heterodimer (“D2-I”) [17,116]. EBV variants deplete SMC5/6, preventing FA-BRCA–mediated DNA repairs and leading to chromosomes with too many centromeres. When mitosis pulls apart multicentromere chromosome structures, the forces shatter the chromosome and induce mutation storms [35]. EBV thus threatens a sprawling, interconnected repair system, including the BRCA pathway, FA proteins, an SMC5/6 scaffold, JAK-STAT signaling, and the SWI-SNF chromatin remodeling complex (Figure 10).

Of course, other environmental, genetic, or lifestyle factors also participate in breast cancer development, but EBV infection exacerbates their effects. Genome deficits in EBV-associated cancers and breast cancers interfere with restoring chromosomes from damage due to natural processes and exogenous mutagens. Some of this damage requires repair pathways that are subject to EBV interference.

Evidence underlying the model in Figure 10 has independent support from the literature. For example, viral load is a marker for the extent of cell-free DNA fragmentation [117]. EBV-mediated transformation routinely generates abnormal karyotypes [118]. The binding of EBNA1 sequence variants increases NPC risk and drives EBV lytic gene expression [119,120], which requires EBV-encoded nucleases [121-123]. Other herpesviruses related to EBV share the ability to fragment DNA and subvert DNA repair pathways [124-126]. EBV facilitates its own replication by interfering with signaling pathways that prevent metastasis [29,127-130]. Independent literature supports EBV participation in metastasis and the results shown in Figure 9. NPC has the highest metastatic rate among all head and neck cancers, and the levels of circulating EBV markers are highly predictive [10]. Finding EBV in lymph nodes of patients with NPC or primary cancer at an unknown site helps detect metastasis [131]. NPC patients with ≥500 copies of EBV per mL plasma had significantly higher rates of liver metastasis than patients with lower EBV levels [132]. EBV-infected B-cells and breast cancer cells both have amplified centrosomes (Figure 10), the mitosis-organizing centers that exert structural control over cell division. The EBV protein thymidine kinase takes up residence in the centrosome [133], and another EBV protein, BNRF1, initiates centrosome amplification in infected B-cells [134]. Overduplication of centrosomes confuses chromatid attachments to spindle fibers during mitosis. Chromosomes do not distribute properly into daughter cells, creating mistakes when the genome replicates [134,135]. Neither centrosome amplification nor chromosome fragmentation (chromothripsis) requires large numbers of viral particles or active infection.

Further bioinformatic tests may still add significant additional information. EBV activation brings massive changes to host chromatin methylation and structure [47,51,136]. Breast cancers have hundreds of these changes [34]. Results here further implicate epigenetic effects, so EBV effects on breast cancer epigenetics should be explored in more detail. EBV is implicated in cancers in multiple additional organs, and the methods developed here may help clarify its potential contributions. Predictions based on virus-human interaction structural biology may also be helpful. The ultimate direct test will be whether childhood recipients of an anti-EBV vaccine have reduced breast cancer incidence. If it even approaches the reduction of cervical cancer achieved by the HPV vaccine (up to 94%), a childhood EBV vaccine could effectively prevent many cases of breast cancer.

### Limitations

EBV itself creates a limitation because the virus can disappear after causing pathogenic genome damage that allows breast cancer to develop. This transitory virus presence forces the use of bioinformatics to look for persistent genome damage EBV leaves behind. EBV disappearance questions whether a group of cancers with EBV connections also contains “sporadic” cancers typed as EBV negative. The EBV-negative forms may have merely lost the criteria used to identify EBV infection, but EBV-related genome damage may still remain. Another limitation is that compared to breast cancers, known EBV-linked cancers such as GC, BL, and NPC are less common, so genome sequence data are also less common.

### Conclusions

In summary, early childhood immunizations against inactivated EBV or selected EBV gene products may significantly reduce the incidence of breast, ovarian, and other cancers, and potentially unexplained chronic diseases. EBV variants lead to DNA breaks, mitotic abnormalities, and the loss of safeguards that protect against breast cancer and its metastasis. Breast cancer breakpoints cluster around breakpoints in EBV cancers, disrupting genes essential to prevent viral infection and breast cancers. A CRISPR-like region on chromosome 6 sequesters some of the thousands of pieces of EBV sequences in the human genome. The same area of chromosome 6 undergoes variable damage in breast cancer, contributing to the reason not everyone with EBV infection develops cancer. In susceptible people, EBV infection leaves behind pathogenic cancer-associated genome abnormalities (“long EBV”). Clinical implications include improvements in evaluating the chances that cancer will return, increased use of immunotherapy for patients with breast cancer that have active infection, and greater urgency in developing an effective EBV vaccine,.

## Supplementary material

10.2196/50712 Multimedia Appendix 1 Glossary and abbreviations.

10.2196/50712 Multimedia Appendix 2 Most of the calculations used in this work.

10.2196/50712 Multimedia Appendix 3 The exact distances between nasopharyngeal cancer and breast cancer breakpoints on chromosome 1. These breaks gather around a few low valleys that periodically occur across the whole chromosome, but the data points are too numerous to display, making the results difficult to interpret.

10.2196/50712 Multimedia Appendix 4 Gene functions at breast cancer breakpoints that clustered around breakpoints in EBV-associated cancers (GC, BL, and NPC), and EBNA1-binding sequences reported in the human genome. BL: Burkitt lymphoma; EBNA1: Epstein-Barr virus nuclear antigen 1; EBV: Epstein-Barr virus; NPC: nasopharyngeal cancer.

10.2196/50712 Multimedia Appendix 5 Alternative explanations.

## Abbreviations

BL: Burkitt lymphoma
BLAST: Basic Local Alignment Search Tool
bp: base pairs
COSMIC: Catalog of Somatic Mutations in Cancer
CRISPR: clustered regularly interspaced short palindromic repeats
DLBCL: diffuse large B-cell lymphoma
EBNA1: Epstein-Barr virus nuclear antigen 1
EBV: Epstein-Barr virus
FA: Fanconi anemia
GC: gastric cancer
HER2: human epidermal growth factor receptor 2
HERV: human endogenous retrovirus
HIV1: human immunodeficiency virus type 1
HLA: human leukocyte antigen
HPV: human papillomavirus
JAK: Janus kinase
MHC: major histocompatibility complex
NF-κB: nuclear factor–κB
NPC: nasopharyngeal cancer
piRNA: Piwi-interacting RNA
SWI-SNF: switch/sucrose non-fermentable
TIL: tumor-infiltrating lymphocyte
