# Supplementary material for: Identifying Safeguards Disabled by Epstein-Barr Virus Infections in Genomes From Patients With Breast Cancer: Chromosomal Bioinformatics Analysis
Source: JMIRx Med. 2025 Jan 29;6:e50712. doi: 10.2196/50712 (PMC11796484; doi:10.2196/50712)
Supplement: Multimedia Appendix 1 [file xmed-v6-e50712-s001.docx]

# Glossary and Abbreviations

| **Abbreviation or term** | **Meaning and explanations** |
| --- | --- |
| **ANXA1** | AnnexinA1, activated by loss of ARID1A [1]. Modulates the immune system to affect the spread of breast cancer. Modulates the effects of estrogen on cell proliferation. |
| **ARID genes** | AT-rich interaction domain. Family of transcription regulators involved in modifying chromatin structures. |
| **BAP1** | BRCA1-associated protein 1, an enzyme that removes ubiquitin groups to modify chromatin during cell cycle progression. |
| **BeAn58058** | A double-stranded DNA virus related to poxviruses and isolated from rodents. |
| **BL** | Burkitt's Lymphoma, an aggressive B-cell lymphoma associated with translocations around the MYC gene locus. |
| **BLAST** | Basic Local Alignment Search Tool, a program and algorithm to compare biological sequence information. |
| **BRCA1** | Breast cancer susceptibility gene 1. |
| **BRCA2** | Breast cancer susceptibility gene 2. |
| **Chromothripsis** | Hundreds to thousands of rearrangements occur in a single event. Tens to hundreds of chromosome breaks occur and are repaired with error-prone methods. |
| **Chr** | Chromosome. |
| **CNTROB** | Centrobin, centriole duplication and spindle assembly protein |
| **COSMIC** | Catalog of Somatic Mutations in Cancer |
| **CRISPR** | Clustered Regularly Interspaced Palindromic Repeats |
| **CTC1** | CST telomere replication complex component 1, protects telomeres from degradation |
| **DLBCL** | Diffuse Large B-Cell Lymphoma. |
| **EBNA1** | The EBV protein that anchors EBV circular DNA to human DNA. |
| **EBV** | Epstein-Barr virus also called human gamma herpesvirus 4 (HHV4). |
| **FA-BRCA pathway** | A complex pathway that regulates DNA repair by homologous recombination. It includes Fanconi anemia genes and BRCA1, BRCA2 genes. |
| **FAM- genes** | [The FAM gene family is a group of genes that have not been fully characterized but encode similar protein sequences. They participate in tumor pathogenesis](https://www.ncbi.nlm.nih.gov/pmc/articles/PMC10235772/#B85). |
| **FASTA** | Text-based format for representing either nucleotide sequences or amino acid (protein) sequences. |
| **FAT3** | Gene for cell adhesion and interactions. Mutations associated with several cancers. |
| **FeLV** | Feline Leukemia Virus, weakens cat immune system, making cats prone to infections and cancer. |
| **GC** | Gastric cancer. |
| **GRCh38** | Genome Reference Consortium Human Genome DNA Sequence Build 38. |
| **hCMV** | Human CytoMegaloVirus, Human herpesvirus 5 (HHV5), a herpesvirus that can be transmitted through the placenta and through breast milk to cause developmental problems in newborns. |
| **HER-2** | Human Epidermal Growth Factor Receptor 2, a protein that regulates cell growth in humans |
| **HERV** | Human Endogenous RetroVirus, ancient retroviruses that have been incorporated into the human genome that may contribute to disease. |
| **HHV8/KSHV** | Kaposi's Sarcoma Virus, causes Kaposi’s sarcoma, a cancer common in AIDS patients |
| **HIV1** | Human Immunodeficiency Virus Type 1 |
| **HKHD40** | An oncogenic virus variant related to EBV |
| **HKNPC60** | Another oncogenic virus variant related to EBV |
| **HLA** | Human Leukocyte Antigen, cell surface proteins that help distinguish self from non-self |
| **Homologous recombination** | An error-free process to repair DNA double strand breaks, crosslinks, and stalled replication intermediates that uses a homologous chromosome as a template to faithfully restore the chromosome. The process requires BRCA1, BRCA2 and Fanconi anemia proteins in the FA-BRCA pathway. |
| **HPV** | Human Papilloma Virus, the cause of most cervical cancer. |
| **HSV-1** | Herpes simplex virus, a common virus that causes painful blisters or ulcers |
| **IFN** | Interferon, a family of anti-viral proteins produced by white blood cells and other cells. Three main types of interferons are termed: interferon-alfa, interferon-beta, and interferon-gamma. Interferons are cytokines and immunomodulators. |
| **JAK-STAT** | The JAK-STAT signaling pathway includes Janus kinases (JAKs), signal transducer and activator of transcription proteins (STATs), and receptors. The pathway relays external chemical signals to the cell nucleus, activating transcription of genes for immunity, cell division, cell death, and tumors. |
| **LCL** | Lymphoblastoid cell line. A cell line created by infecting whole blood with EBV to preserve B-cells. |
| **LUZP2** | Leucine zipper protein significantly upregulated in some cancers. |
| **MHC** | major histocompatibility complex, a group of genes encoding proteins with key roles in the immune system. |
| **MMTV** | Mouse mammary tumor virus, milk transmitted retrovirus that causes most mammary tumors in mice. |
| **MYC** | A proto-oncogene encoding a nuclear phosphoprotein involved in cell cycle progression, apoptosis and cellular transformation. |
| **NCBI** | National Center for Biotechnology Information. |
| **NFKB** | family of transcription factors responding to a variety of immune stimulating cytokines also important for development of immune T- and B- lymphocytes. |
| **NPC** | Nasopharyngeal cancer, cancer at the back of the throat behind the nose. |
| **PBRM1** | Protein Polybromo 1, a component of the SWI-SNF chromatin remodeling complex. |
| **PERV** | Porcine Endogenous Retrovirus, present in pigs and capable of infecting humans. |
| **PIK3CA** | A gene encoding a catalytic subunit of a protein that phosphorylates certain signaling molecules. |
| **piRNAs** | Piwi-interacting RNAs are non-coding RNAs composed of 24–32 nucleotides. |
| **SSA** | Single strand annealing, a method of DNA repair. |
| **SMARCE1** | SWI-SNF related, matrix associated, actin dependent. One component of the SWI-SNF complex which repositions nucleosomes along DNA. |
| **SWI-SNF** | SWI-SNF (SWItch/Sucrose Non-Fermentable) group of ATP-dependent proteins that associate to remodel how DNA is packaged in chromatin. |
| **TIL** | Tumor-infiltrating lymphocytes. |
| **TP53** | A tumor suppressor gene encoding a protein regulating cell division and death. |
| **transposon** | A nucleic acid sequence in DNA that can change its position in the genome, sometimes creating mutations. Many transposons have been inactivated. |
| **VHL** | Von Hippel-Lindau gene, a tumor suppressor gene that regulates cell growth and division. |

References:

1. Berns K, Sonnenblick A, Gennissen A, et al. Loss of ARID1A activates ANXA1, which serves as a predictive biomarker for trastuzumab resistance. Clin Cancer Res. Nov 1, 2016;22(21):5238-5248.
