# Supplementary figures and images for: Identifying Safeguards Disabled by Epstein-Barr Virus Infections in Genomes From Patients With Breast Cancer: Chromosomal Bioinformatics Analysis
Source: JMIRx Med. 2025 Jan 29;6:e50712. doi: 10.2196/50712 (PMC11796484; doi:10.2196/50712)

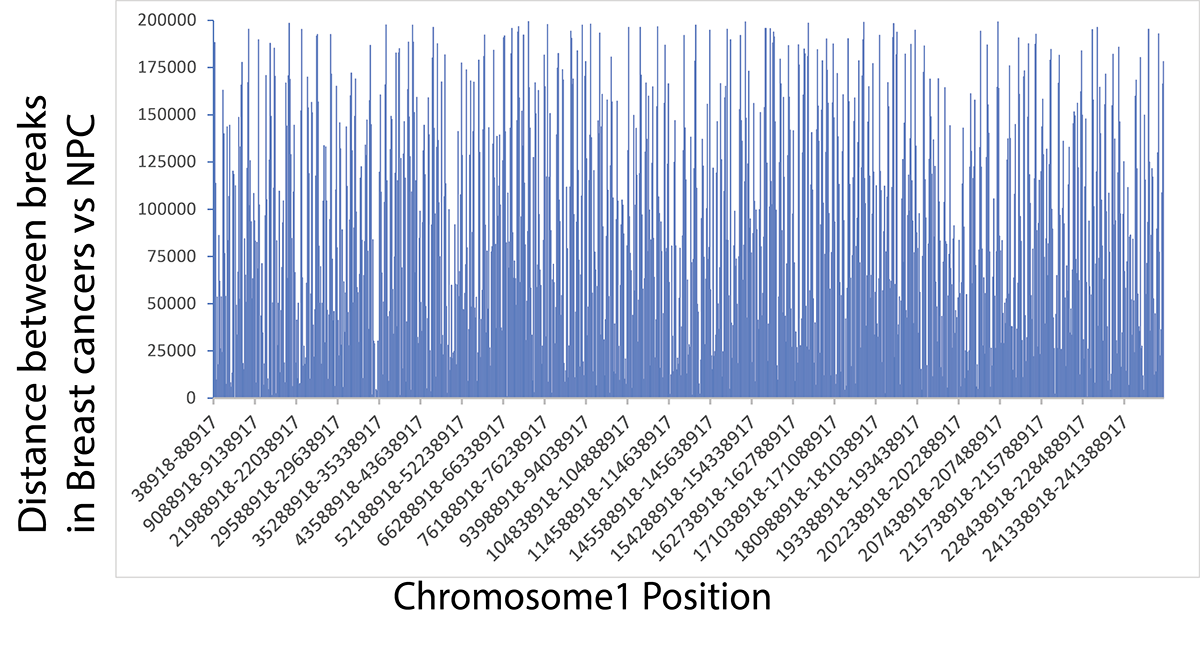

Supplement: Multimedia Appendix 3 [file xmed-v6-e50712-s003.png]

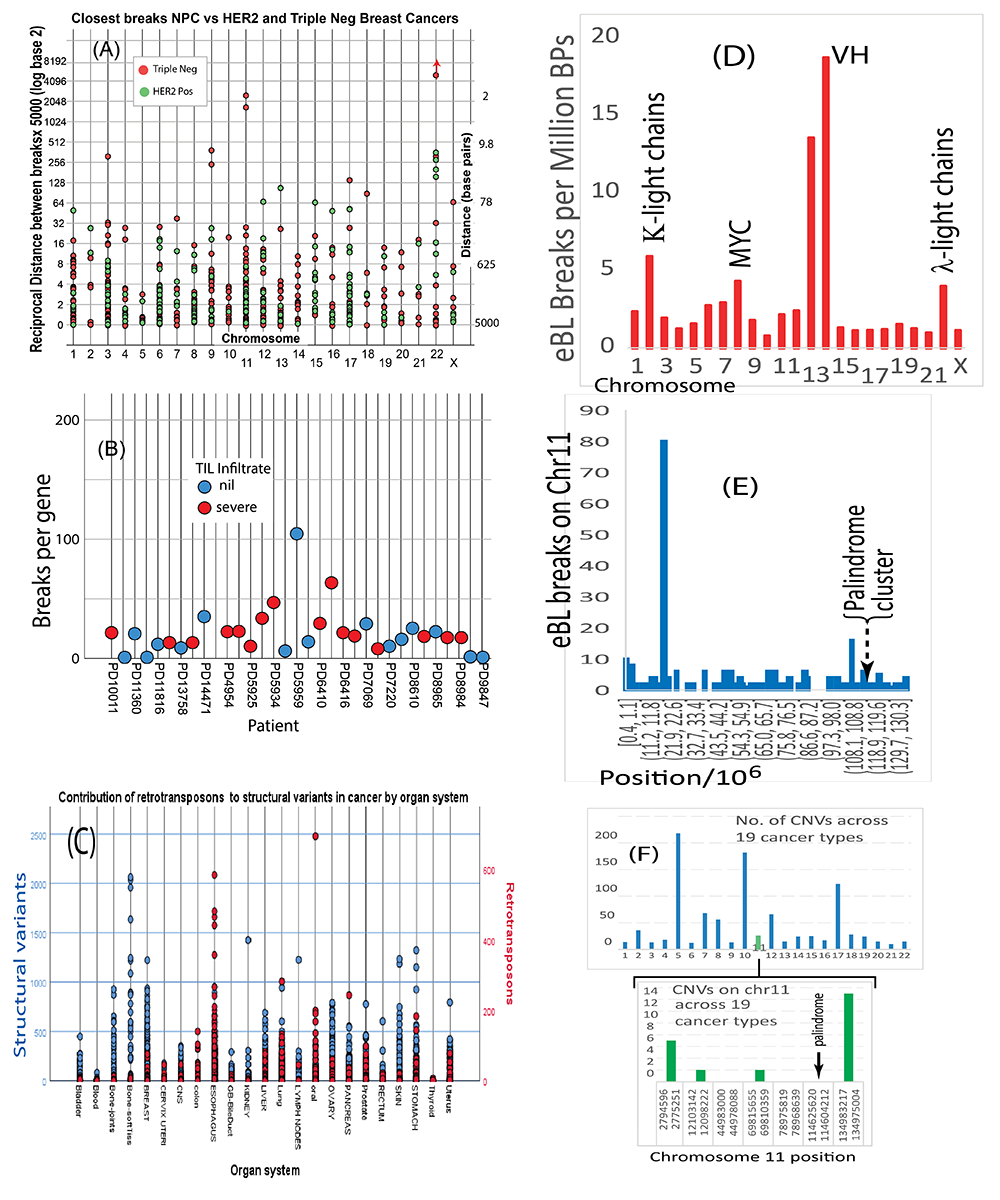

Supplement: Multimedia Appendix 5 [file xmed-v6-e50712-s005.png]
