# Supplementary material for: Identifying Safeguards Disabled by Epstein-Barr Virus Infections in Genomes From Patients With Breast Cancer: Chromosomal Bioinformatics Analysis
Source: JMIRx Med. 2025 Jan 29;6:e50712. doi: 10.2196/50712 (PMC11796484; doi:10.2196/50712)
Supplement: Multimedia Appendix 4 [file xmed-v6-e50712-s004.docx]

| Table S1: Gene functions at breast cancer breakpoints clustered around breakpoints in EBV-associated cancers GC, BL, and NPC | | | | |
| --- | --- | --- | --- | --- |
| Gene | **Chromosome** | **Approximate Breakpoint Regions** | **Function encoded from NIH gene database** | **Expression in breast /ovarian cancer** |
| serpins | 6 | 1-5,000,000 | Protease inhibitors that control inflammation and coagulation | Overexpression of SerpinA3 promotes tumor invasion [1] |
| IRF4 | 6 | 1-5,000,000 | Antiviral defense | Re-expression inhibits breast cancer cells [2] |
| WRNIP1 | 6 | 1-5,000,000 | DNA damage response related to the FA-BRCA pathway | Interaction with a translocated tyrosine receptor kinase promotes breast cancer survival and metastasis [3] |
| MHC genes | 6 | Around 30,000,000 | Cell surface proteins involved in immune recognition of foreign proteins | Class 1 downregulates HER2 . Class II associated with good prognosis[4, 5] |
| ARGEF3 | 6 | 137,500,000 | May enhance estrogen receptor activity in breast cancer cells. | Overexpression Is unfavorable (proteinatlas.org) |
| PERP | 6 | 137,500,000 | Apoptosis effector | Inhibition promotes breast cancer [6] |
| TNFAIP3 | 6 | 137,500,000 | NFKB inhibitor, inhibits inflammation | Promotes breast cancer inflammation and metastasis [7] |
| FBXO2 | 8 | 1-5,000,000 | Tumor suppressor | Down-regulated[8] |
| ERBB2 | 8 | 37,000,000-42,000,000 | Receptor tyrosine kinase that regulates extracellular matrix components, cell mobility | Frequently amplified as part of a breast cancer amplicon [9] |
| Intermediate filaments (IFs) | 8 | 37,000,000-42,000,000 | Often associate with cancer progression, cell migration and invasion [61] | 73 genes may increase or decrease breast carcinoma growth [10] |
| ASHL2 | 8 | 37,000,000-42,000,000 | Histone methyltransferase complex subunit | Breast cancer cell invasion and migration [11] |
| ADAM genes | 8 | 37,000,000-42,000,000 | T-cell response to acute EBV infection [62] | ADAM28 is overexpressed [12] |
| PAG1 | 8 | 80,000,000 | T-cell receptor, EGFR signaling | Upregulation promotes progression and chemoresistance [13] |
| PDP1 | 8 | 90,000,000 | Energy production | Promotes progression [14] |
| ESRP1 | 8 | 90,000,000 | Alternate splicing | Overexpressed, high levels associate with poor prognosis [15] |
| CCNE2 | 8 | 90,000,000 | Cell cycle regulation | Amplified as part of the 8q22 locus [16] |
| TP53INP1 | 8 | 90,000,000 | Tumor suppressor that stimulates TP53 | Low expression predicts poor prognosis[17] |
| POLR | 8 | 100,000,000 | Host cell transcription | Subunit regulates tumorigenesis and metastasis [18] |
| COX6C | 8 | 100,000,000 | Energy production | Mutations may influence breast cancer [19] |
| MYC | 8 | 128,000,000 | Promotes transcription | Overexpressed [20] |
| CHRAC1 | 8 | 140,000,000 | Chromatin accessibility | Enhances tumor growth [21] |
| RELA,RAB1B | 11 | 66,000,000 | Oncogenes | RELA overexpressed in TNBC [22]  RAB1B loss promotes TNBC metastasis [23] |
| FRA11A | 11 | 66,000,000 | Fragile site that affects hypermethylation | Flanks genes that are coamplified in breast cancer [24] |
| KDM2A, KDM2B | 11 | 66,000,000 | Lysine methyltransferases | KDM2A promotes invasion and metastasis [25]. KDM2B is overexpressed and promotes TNBC [26] |
| CCND1 | 11 | 66,000,000 | Cell cycle control | Aberrant expression in breast cancer [27] |
| FADD | 11 | 66,000,000 | Cell death domain | Weak expression associated with invasive breast cancer [28] |
| CNTROB | 17 | 7,000,000-10,000,000 | Centriole duplication and spindle assembly, Interacts with BRCA2 required for centriole function. | Centrosome amplification associates with high grade breast cancer [29]. Excess CNTROB expression [30] |
| tRNA’s | 17 | 7,000,000-10,000,000 | Transfer RNAs | Upregulated [31] |
| CTC1 | 17 | 7,000,000-10,000,000 | CST telomere replication complex component 1. Telomere maintenance | Part of a complex amplified in breast cancer [32] |
| MED9 | 17 | 17,000,000-20,000,000 | Mediator complex subunit 9. Required to activate RNA polymerase II | Mediator subunits contribute to estrogen mediated gene regulation in breast cancer [33] |
| SMARCE1 | 17 | 40,000,000-45,000,000 | SWI-SNF related, actin dependent regulator of chromatin, subfamily e, member 1 | High expression is associated with relapse [34] |
| BRCA1 | 17 | 40,000,000-45,000,000 | Breast cancer predisposition, Homologous recombination repair, genome stability | Expression level and subcellular location are prognostic markers [35] |
| FAAP100 | 17 | 81538566-51554456 | Component of the Fanconi anemia core complex, required for core complex stability and FANCD2 mono-ubiquitination | The core complex is tequired for activity of BRCA1 and BRCA2 |

References for Table S1:

1. Zhang Y, Tian J, Qu C, Peng Y, Lei J, Li K, et al. Overexpression of SERPINA3 promotes tumor invasion and migration, epithelial-mesenchymal-transition in triple-negative breast cancer cells. Breast Cancer. 2021 Jul;28(4):859-73. PMID: 33569740. doi: 10.1007/s12282-021-01221-4.

2. Vernier M, McGuirk S, Dufour CR, Wan L, Audet-Walsh E, St-Pierre J, et al. Inhibition of DNMT1 and ERRalpha crosstalk suppresses breast cancer via derepression of IRF4. Oncogene. 2020 Oct;39(41):6406-20. PMID: 32855526. doi: 10.1038/s41388-020-01438-1.

3. Marquez-Palencia M, Reza Herrera L, Parida PK, Ghosh S, Kim K, Das NM, et al. AXL/WRNIP1 Mediates Replication Stress Response and Promotes Therapy Resistance and Metachronous Metastasis in HER2+ Breast Cancer. Cancer research. 2024 Mar 4;84(5):675-87. PMID: 38190717. doi: 10.1158/0008-5472.CAN-23-1459.

4. Inoue M, Mimura K, Izawa S, Shiraishi K, Inoue A, Shiba S, et al. Expression of MHC Class I on breast cancer cells correlates inversely with HER2 expression. Oncoimmunology. 2012 Oct 1;1(7):1104-10. PMID: 23170258. doi: 10.4161/onci.21056.

5. Forero A, Li Y, Chen D, Grizzle WE, Updike KL, Merz ND, et al. Expression of the MHC Class II Pathway in Triple-Negative Breast Cancer Tumor Cells Is Associated with a Good Prognosis and Infiltrating Lymphocytes. Cancer Immunol Res. 2016 May;4(5):390-9. PMID: 26980599. doi: 10.1158/2326-6066.CIR-15-0243.

6. Shan BQ, Wang XM, Zheng L, Han Y, Gao J, Lv MD, et al. DCAF13 promotes breast cancer cell proliferation by ubiquitin inhibiting PERP expression. Cancer science. 2022 May;113(5):1587-600. PMID: 35178836. doi: 10.1111/cas.15300.

7. Song C, Kendi AT, Lowe VJ, Lee S. The A20/TNFAIP3-CDC20-CASP1 Axis Promotes Inflammation-mediated Metastatic Disease in Triple-negative Breast Cancer. Anticancer Res. 2022 Feb;42(2):681-95. PMID: 35093867. doi: 10.21873/anticanres.15527.

8. Liu Y, Pan B, Qu W, Cao Y, Li J, Zhao H. Systematic analysis of the expression and prognosis relevance of FBXO family reveals the significance of FBXO1 in human breast cancer. Cancer Cell Int. 2021 Feb 23;21(1):130. PMID: 33622332. doi: 10.1186/s12935-021-01833-y.

9. Kauraniemi P, Kallioniemi A. Activation of multiple cancer-associated genes at the ERBB2 amplicon in breast cancer. Endocrine-related cancer. 2006 Mar;13(1):39-49. PMID: 16601278. doi: 10.1677/erc.1.01147.

10. Sharma P, Alsharif S, Fallatah A, Chung BM. Intermediate Filaments as Effectors of Cancer Development and Metastasis: A Focus on Keratins, Vimentin, and Nestin. Cells. 2019 May 23;8(5). PMID: 31126068. doi: 10.3390/cells8050497.

11. Batbayar G, Ishimura A, Lyu H, Wanna-Udom S, Meguro-Horike M, Terashima M, et al. ASH2L, a COMPASS core subunit, is involved in the cell invasion and migration of triple-negative breast cancer cells through the epigenetic control of histone H3 lysine 4 methylation. Biochemical and biophysical research communications. 2023 Aug 20;669:19-29. PMID: 37262949. doi: 10.1016/j.bbrc.2023.05.061.

12. Mitsui Y, Mochizuki S, Kodama T, Shimoda M, Ohtsuka T, Shiomi T, et al. ADAM28 is overexpressed in human breast carcinomas: implications for carcinoma cell proliferation through cleavage of insulin-like growth factor binding protein-3. Cancer research. 2006 Oct 15;66(20):9913-20. PMID: 17047053. doi: 10.1158/0008-5472.CAN-06-0377.

13. Lu Y, Yang Y, Liu Y, Hao Y, Zhang Y, Hu Y, et al. Upregulation of PAG1/Cbp contributes to adipose-derived mesenchymal stem cells promoted tumor progression and chemoresistance in breast cancer. Biochemical and biophysical research communications. 2017 Dec 16;494(3-4):719-27. PMID: 29079189. doi: 10.1016/j.bbrc.2017.10.118.

14. Song Y, Zhang J, Zhang L, Zhang S, Shen C. PDP1 Promotes Cell Malignant Behavior and Is Associated with Worse Clinical Features in Ovarian Cancer Patients: Evidence from Bioinformatics and In Vitro Level. Comput Math Methods Med. 2022;2022:7397250. PMID: 36276992. doi: 10.1155/2022/7397250.

15. Gokmen-Polar Y, Neelamraju Y, Goswami CP, Gu Y, Gu X, Nallamothu G, et al. Splicing factor ESRP1 controls ER-positive breast cancer by altering metabolic pathways. EMBO Rep. 2019 Feb;20(2). PMID: 30665944. doi: 10.15252/embr.201846078.

16. Milioli HH, Alexandrou S, Lim E, Caldon CE. Cyclin E1 and cyclin E2 in ER+ breast cancer: prospects as biomarkers and therapeutic targets. Endocrine-related cancer. 2020 May;27(5):R93-R112. PMID: 32061162. doi: 10.1530/ERC-19-0501.

17. Nishimoto M, Nishikawa S, Kondo N, Wanifuchi-Endo Y, Hato Y, Hisada T, et al. Prognostic impact of TP53INP1 gene expression in estrogen receptor alpha-positive breast cancer patients. Jpn J Clin Oncol. 2019 Jun 1;49(6):567-75. PMID: 30855679. doi: 10.1093/jjco/hyz029.

18. Lautre W, Richard E, Feugeas JP, Dumay-Odelot H, Teichmann M. The POLR3G Subunit of Human RNA Polymerase III Regulates Tumorigenesis and Metastasis in Triple-Negative Breast Cancer. Cancers. 2022 Nov 22;14(23). PMID: 36497214. doi: 10.3390/cancers14235732.

19. de Oliveira RC, Dos Reis SP, Cavalcante GC. Mutations in Structural Genes of the Mitochondrial Complex IV May Influence Breast Cancer. Genes (Basel). 2023 Jul 18;14(7). PMID: 37510369. doi: 10.3390/genes14071465.

20. Xu J, Chen Y, Olopade OI. MYC and Breast Cancer. Genes Cancer. 2010 Jun;1(6):629-40. PMID: 21779462. doi: 10.1177/1947601910378691.

21. Li S, Wang L, Shi J, Chen Y, Xiao A, Huo B, et al. Chromatin accessibility complex subunit 1 enhances tumor growth by regulating the oncogenic transcription of YAP in breast and cervical cancer. PeerJ. 2024;12:e16752. PMID: 38223760. doi: 10.7717/peerj.16752.

22. Kanzaki H, Chatterjee A, Hossein Nejad Ariani H, Zhang X, Chung S, Deng N, et al. Disabling the Nuclear Translocalization of RelA/NF-kappaB by a Small Molecule Inhibits Triple-Negative Breast Cancer Growth. Breast Cancer (Dove Med Press). 2021;13:419-30. PMID: 34262338. doi: 10.2147/BCTT.S310231.

23. Jiang HL, Sun HF, Gao SP, Li LD, Hu X, Wu J, et al. Loss of RAB1B promotes triple-negative breast cancer metastasis by activating TGF-beta/SMAD signaling. Oncotarget. 2015 Jun 30;6(18):16352-65. PMID: 25970785. doi: 10.18632/oncotarget.3877.

24. Coquelle A, Pipiras E, Toledo F, Buttin G, Debatisse M. Expression of fragile sites triggers intrachromosomal mammalian gene amplification and sets boundaries to early amplicons. Cell. 1997 Apr 18;89(2):215-25. PMID: 9108477. doi: 10.1016/s0092-8674(00)80201-9.

25. Liu L, Liu J, Lin Q. Histone demethylase KDM2A: Biological functions and clinical values (Review). Exp Ther Med. 2021 Jul;22(1):723. PMID: 34007332. doi: 10.3892/etm.2021.10155.

26. Chavdoula E, Anastas V, La Ferlita A, Aldana J, Carota G, Spampinato M, et al. Transcriptional regulation of amino acid metabolism by KDM2B, in the context of ncPRC1.1 and in concert with MYC and ATF4. Metabolism: clinical and experimental. 2024 Jan;150:155719. PMID: 37935302. doi: 10.1016/j.metabol.2023.155719.

27. Wang J, Su W, Zhang T, Zhang S, Lei H, Ma F, et al. Aberrant Cyclin D1 splicing in cancer: from molecular mechanism to therapeutic modulation. Cell death & disease. 2023 Apr 6;14(4):244. PMID: 37024471. doi: 10.1038/s41419-023-05763-7.

28. Marin-Rubio JL, Vela-Martin L, Fernandez-Piqueras J, Villa-Morales M. FADD in Cancer: Mechanisms of Altered Expression and Function, and Clinical Implications. Cancers. 2019 Sep 29;11(10). PMID: 31569512. doi: 10.3390/cancers11101462.

29. Denu RA, Zasadil LM, Kanugh C, Laffin J, Weaver BA, Burkard ME. Centrosome amplification induces high grade features and is prognostic of worse outcomes in breast cancer. BMC cancer. 2016 Jan 29;16:47. PMID: 26832928. doi: 10.1186/s12885-016-2083-x.

30. Parizi aK, Seresht LM, Esmaeili S-A, Jazi AD, Sarli A, Khosravian F, et al. Evaluation of the association between centrosome amplification in tumor tissue of breast cancer patients and changes in the expression of CETN1 and CNTROB genes. Gene reports. 2022;26. doi: <https://doi.org/10.1016/j.genrep.2021.101481>.

31. Zhang Z, Ye Y, Gong J, Ruan H, Liu CJ, Xiang Y, et al. Global analysis of tRNA and translation factor expression reveals a dynamic landscape of translational regulation in human cancers. Commun Biol. 2018;1:234. PMID: 30588513. doi: 10.1038/s42003-018-0239-8.

32. Wang L, Ma T, Liu W, Li H, Luo Z, Feng X. Pan-Cancer Analyses Identify the CTC1-STN1-TEN1 Complex as a Protective Factor and Predictive Biomarker for Immune Checkpoint Blockade in Cancer. Front Genet. 2022;13:859617. PMID: 35368664. doi: 10.3389/fgene.2022.859617.

33. Weber H, Garabedian MJ. The mediator complex in genomic and non-genomic signaling in cancer. Steroids. 2018 May;133:8-14. PMID: 29157917. doi: 10.1016/j.steroids.2017.11.007.

34. Sokol ES, Feng YX, Jin DX, Tizabi MD, Miller DH, Cohen MA, et al. SMARCE1 is required for the invasive progression of in situ cancers. Proceedings of the National Academy of Sciences of the United States of America. 2017 Apr 18;114(16):4153-8. PMID: 28377514. doi: 10.1073/pnas.1703931114.

35. Mahmoud AM, Macias V, Al-Alem U, Deaton RJ, Kadjaksy-Balla A, Gann PH, et al. BRCA1 protein expression and subcellular localization in primary breast cancer: Automated digital microscopy analysis of tissue microarrays. PloS one. 2017;12(9):e0184385. PMID: 28863181. doi: 10.1371/journal.pone.0184385.

### Table S2. EBNA1 binding sequences reported in the human genome

|  | Position in human DNA sequence that binds the EBV anchor protein EBNA1 | | | | | | | | | | | | | | | | | | | | | | | | | | | | | |
| --- | --- | --- | --- | --- | --- | --- | --- | --- | --- | --- | --- | --- | --- | --- | --- | --- | --- | --- | --- | --- | --- | --- | --- | --- | --- | --- | --- | --- | --- | --- |
| Ref | 1 | 2 | 3 | 4 | 5 | 6 | 7 | 8 | 9 | 10 | 11 | 12 | 13 | 14 | 15 | 16 | 17 | 18 | 19 | 20 | 21 | 22 | 23 | 24 | 25 | 26 | 27 | 28 | 29 | 30 |
| [1] | G | G | G | T | A | G | C | A | T | A | T | G | C | T | A | C | C | C |  |  |  |  |  |  |  |  |  |  |  |  |
| [2] | G | G | G | T | A | A | C | C | A | C/G/T | T | G | T | T | A | C | C | C/T |  |  |  |  |  |  |  |  |  |  |  |  |
| [3] | G/A | G | G | C/T/A | A | G | C/T | A | C/T | A/T | T | G/A | C | T | A/G/T | C/T | C |  |  |  |  |  |  |  |  |  |  |  |  |  |
| [3] | T | G | G | A | T | A | A | T | A | A | G | T | G/A | T | T | G | C | C | T | C | G/T/A | T/A | G | G | G | T | A | A | C/T/A | C |
| [3] | G/T/A | T/G | G/A | T | G/T | T | G | T/A | G/A/C | T/G | G/T | T/C/A | G/A |  |  |  |  |  |  |  |  |  |  |  |  |  |  |  |  |  |
| [3] | G | G/A | A/G | T/C | T/G/C | A/C/T | C/T | A/T/C | G/A | G/A | C/T/A | A/G/C | T/C | G/C | A/C | G/C | C/G | C | A/G/C | C/A/T | C/T | A/G/C | T/C | G/A/T | C/T/G | C/T | C/T/G | A/G/C | C/T/C | C |
| [3] | C | T/A/C | CT/G | C/T | T/A | C/G | C | T/A | C/G | C | A/T/C |  |  |  |  |  |  |  |  |  |  |  |  |  |  |  |  |  |  |  |

### References for Table S2:

1. Bochkarev A, Barwell JA, Pfuetzner RA, Bochkareva E, Frappier L, Edwards AM. Crystal structure of the DNA-binding domain of the Epstein-Barr virus origin-binding protein, EBNA1, bound to DNA. Cell. 1996 Mar 8;84(5):791-800. PMID: 8625416. doi: 10.1016/s0092-8674(00)81056-9.

2. Li JSZ, Abbasi A, Kim DH, Lippman SM, Alexandrov LB, Cleveland DW. Chromosomal fragile site breakage by EBV-encoded EBNA1 at clustered repeats. Nature. 2023 Apr;616(7957):504-9. PMID: 37046091. doi: 10.1038/s41586-023-05923-x.

3. Lu F, Wikramasinghe P, Norseen J, Tsai K, Wang P, Showe L, et al. Genome-wide analysis of host-chromosome binding sites for Epstein-Barr Virus Nuclear Antigen 1 (EBNA1). Virology journal. 2010;7:262. PMID: 20929547. doi: 10.1186/1743-422X-7-262.
